# Cancer-Associated Fibroblasts Are Key Determinants of Cancer Cell Invasion in the Earliest Stage of Colorectal Cancer

**DOI:** 10.1016/j.jcmgh.2023.04.004

**Published:** 2023-04-20

**Authors:** Hao Dang, Tom J. Harryvan, Chen-Yi Liao, Erik H.J. Danen, Vienna N.L.N. Spalburg, Szymon M. Kielbasa, Hailiang Mei, Jelle J. Goeman, Eveline S. de Jonge-Muller, Stefanus G.T. Janson, Johan J. van der Reijden, Stijn Crobach, James C.H. Hardwick, Jurjen J. Boonstra, Noel F.C.C. de Miranda, Lukas J.A.C. Hawinkels

**Affiliations:** 1 Department of Gastroenterology and Hepatology, Leiden University Medical Center, Leiden, The Netherlands; 2 Division of Drug Discovery and Safety, Leiden Academic Centre of Drug Research, Leiden University, Leiden, The Netherlands; 3 Department of Biomedical Data Sciences, Leiden University Medical Center, Leiden, The Netherlands; 4 Department of Pathology, Leiden University Medical Center, Leiden, The Netherlands

**Keywords:** T1 Colorectal Cancer, Tumor Microenvironment, Cancer-Associated Fibroblast

## Abstract

**Background & Aims:**

Improving clinical management of early stage colorectal cancers (T1CRCs) requires a better understanding of their underlying biology. Accumulating evidence shows that cancer-associated fibroblasts (CAFs) are important determinants of tumor progression in advanced colorectal cancer (CRC), but their role in the initial stages of CRC tumorigenesis is unknown. Therefore, we investigated the contribution of T1CAFs to early CRC progression.

**Methods:**

Primary T1CAFs and patient-matched normal fibroblasts (NFs) were isolated from endoscopic biopsy specimens of histologically confirmed T1CRCs and normal mucosa, respectively. The impact of T1CAFs and NFs on tumor behavior was studied using 3-dimensional co-culture systems with primary T1CRC organoids and extracellular matrix (ECM) remodeling assays. Whole-transcriptome sequencing and gene silencing were used to pinpoint mediators of T1CAF functions.

**Results:**

In 3-dimensional multicellular cultures, matrix invasion of T1CRC organoids was induced by T1CAFs, but not by matched NFs. Enhanced T1CRC invasion was accompanied by T1CAF-induced ECM remodeling and up-regulation of CD44 in epithelial cells. RNA sequencing of 10 NF-T1CAF pairs revealed 404 differentially expressed genes, with significant enrichment for ECM-related pathways in T1CAFs. Cathepsin H, a cysteine-type protease that was specifically up-regulated in T1CAFs but not in fibroblasts from premalignant lesions or advanced CRCs, was identified as a key factor driving matrix remodeling by T1CAFs. Finally, we showed high abundance of cathepsin H–expressing T1CAFs at the invasive front of primary T1CRC sections.

**Conclusions:**

Already in the earliest stage of CRC, cancer cell invasion is promoted by CAFs via direct interactions with epithelial cancer cells and stage-specific, cathepsin H–dependent ECM remodeling. RNA sequencing data of the 10 NF-T1CAF pairs can be found under GEO accession number GSE200660.

## Graphical abstract

**Figure:**
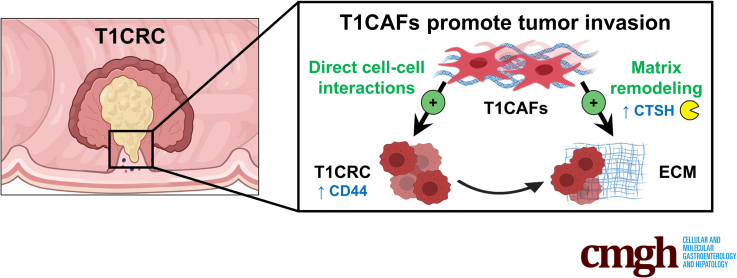

**Summary**

Already in the earliest stage of invasive colorectal cancer, cancer-associated fibroblasts in T1 colorectal cancer promote cancer cell invasion via direct cell–cell interactions with epithelial T1 colorectal cancer cells and increased matrix remodeling. The latter depended on stage-specific up-regulation of cathepsin H in T1 colorectal cancer-associated fibroblasts.

Colorectal cancer (CRC) is one of the most frequently diagnosed malignancies in the world.^1^ Most CRCs develop via the adenoma–carcinoma sequence, a process that involves the progression of precursor lesions, adenomatous polyps, into invasive cancers with metastatic potential. Historically, CRCs often were detected at an advanced symptomatic stage, but the introduction of population-based screening has greatly increased the detection of CRC at earlier stages.^2^ This has opened avenues for local organ-preserving treatments, but also posed new challenges in clinical management.

Currently, one of the most clinically challenging dilemmas involves submucosally invasive CRCs (T1CRCs). T1CRCs are in the earliest stage of invasiveness and often can be cured by organ-preserving endoscopic resection because of their indolent tumor behavior.^3^ Only a small proportion (∼10%) of T1CRCs also display lymph node metastasis and require oncological surgery after the initial organ-preserving treatment.^4^ However, in current practice it remains very difficult to accurately distinguish between aggressive and indolent T1CRCs, resulting in >80% surgical resections without additional oncological benefit.^4^ This warrants the need for a better understanding of the underlying tumor biology of T1CRCs.

Cancers often are described as “wounds that do not heal” because of the similarities of biological processes that occur in wound healing and tumorigenesis.^5^ In both settings, a key event is the activation of fibroblasts in reaction to an injury stimulus. Activated fibroblasts adopt functions that are beneficial to tissue repair, such as increased production of extracellular matrix (ECM) proteins, matrix remodeling enzymes or cytokines that stimulate cell proliferation.^6^ In a normal wound healing response, the activated state of fibroblasts is reverted upon restoration of tissue integrity. However, this resolution phase becomes disrupted during malignant progression, gradually giving rise to cancer-associated fibroblasts (CAFs). CAFs is an umbrella term for all fibroblasts within and surrounding a tumor.^7^ Numerous studies have shown that CAFs are critical determinants of CRC biology and clinical outcome,^6^^,^^8^, ^9^, ^10^ but it should be noted that this knowledge mainly comes from work on advanced CRCs. Given the gradual nature of the changes in fibroblast populations during cancer development,^10^, ^11^, ^12^ it remains to be elucidated to what extent CAFs in early CRC stages already contribute to tumor progression.

Our current study comprehensively addresses the function and phenotype of CAFs in the earliest stage of CRC invasion: T1 tumors. Using a large biobank of patient-derived, normal-tumor fibroblast pairs, we discovered that CAFs in T1CRC (T1CAFs) show considerable phenotypic differences from patient-matched normal fibroblasts (NFs), including stage-specific traits that are not recapitulated in fibroblasts derived from premalignant polyps or more advanced CRCs. Using primary 3-dimensional (3D) organoid-fibroblast co-cultures and microinjection-based ECM remodeling assays, we found that T1CAFs are key regulators of early stage cancer cell invasion and show increased matrix remodeling capacity compared with matched NFs. Next, through unbiased transcriptomic profiling we uncovered cathepsin H as a novel T1 stage–specific mediator of T1CAF-induced matrix remodeling. Finally, we validated the relative abundance of cathepsin H–expressing T1CAFs on primary T1CRC sections, thereby highlighting the translational potential of our findings.

## Results

### T1CAFs Promote T1CRC Invasion Through Basement Membrane Proteins

**Figure 1:**
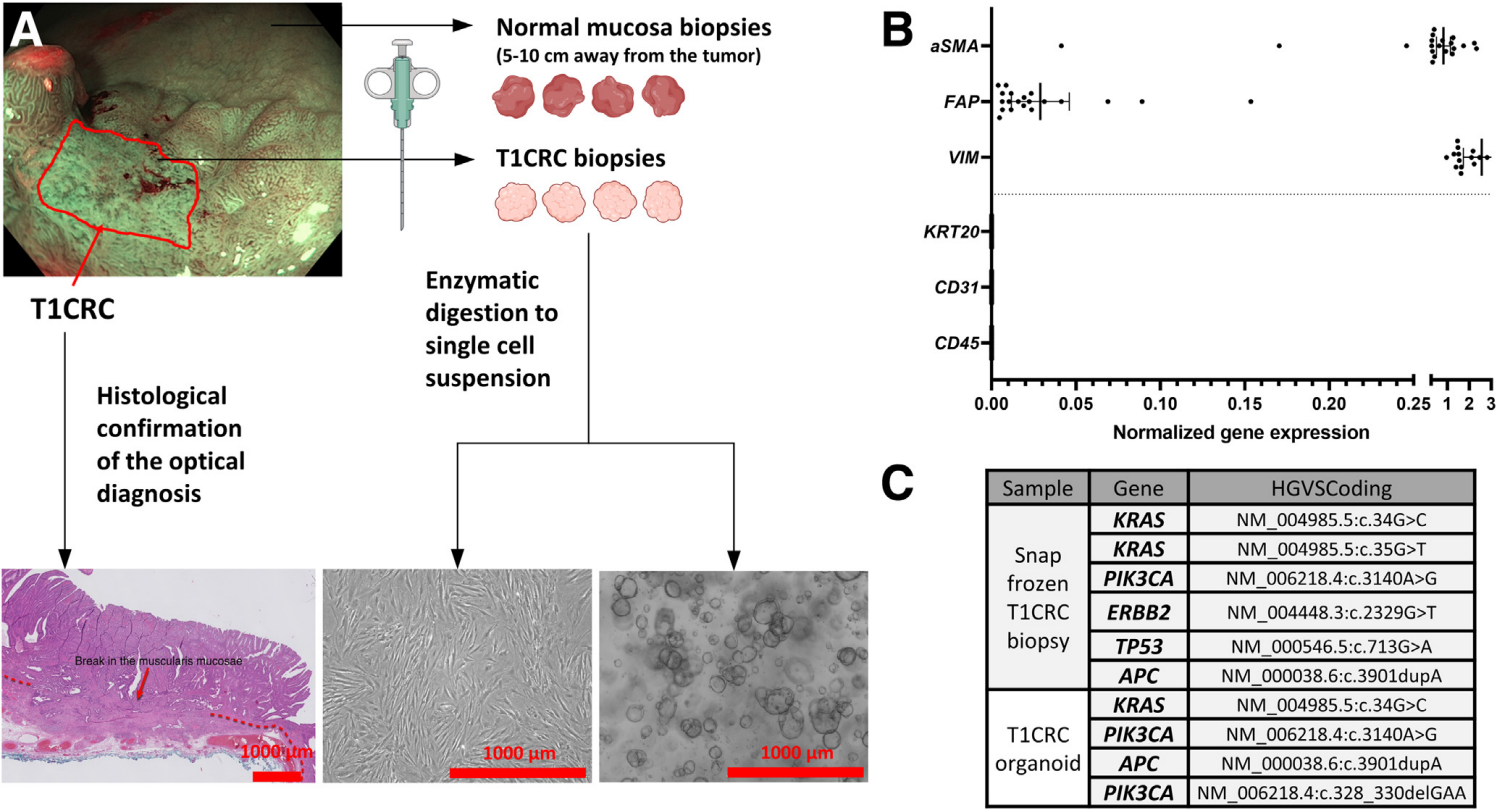
**Isolation and validation of primary NF-T1CAF pairs and T1CRC organoids.** (*A*) Workflow of fibroblast isolation. (*B*) Gene expression levels of fibroblast markers (*αSMA*, *FAP*, *VIM*) and negative selection markers (*KRT20*, epithelial cells; *CD31*, endothelial cells; *CD45*, immune cells) in primary NFs and T1CAFs subjected to functional experiments (n = 10 pairs). (*C*) DNA sequencing of isolated primary T1CRC organoids and the original T1CRC biopsy specimens. HGVS, Human Genome Variation Society.

To study T1CAF biology, we cultured patient-matched T1CAFs and NFs from endoscopic biopsy specimens of T1CRC and matched normal mucosa (Figure 1*A*). Several biopsy specimens were taken from regions with optical features of T1CRC (subsequently confirmed on histology), and from normal adjacent tissue 5–10 cm away from the tumor. To confirm the fibroblast origin of the isolated fibroblast lines, we used a quantitative polymerase chain reaction (qPCR) panel of positive and negative selection markers. High expression levels of fibroblast marker genes (*αSMA*, *FAP*, and *VIM*) and the absence of epithelial (*KRT20*), immune (*CD45*) and endothelial cell (*CD31*) marker expression were shown in the NF-T1CAF pairs that were subjected to further functional experiments (Figure 1*B*). Parallel to T1CAF isolation, we also isolated primary T1CRC organoids from endoscopic T1CRC biopsy specimens according to standard procedures^13^ (Figure 1*A*). DNA sequencing confirmed that these organoids harbored mutations in CRC genes (*APC*, *KRAS*, and *PIK3CA*) that corresponded to the mutations found in the original tumor (Figure 1*C*).

**Figure 2:**
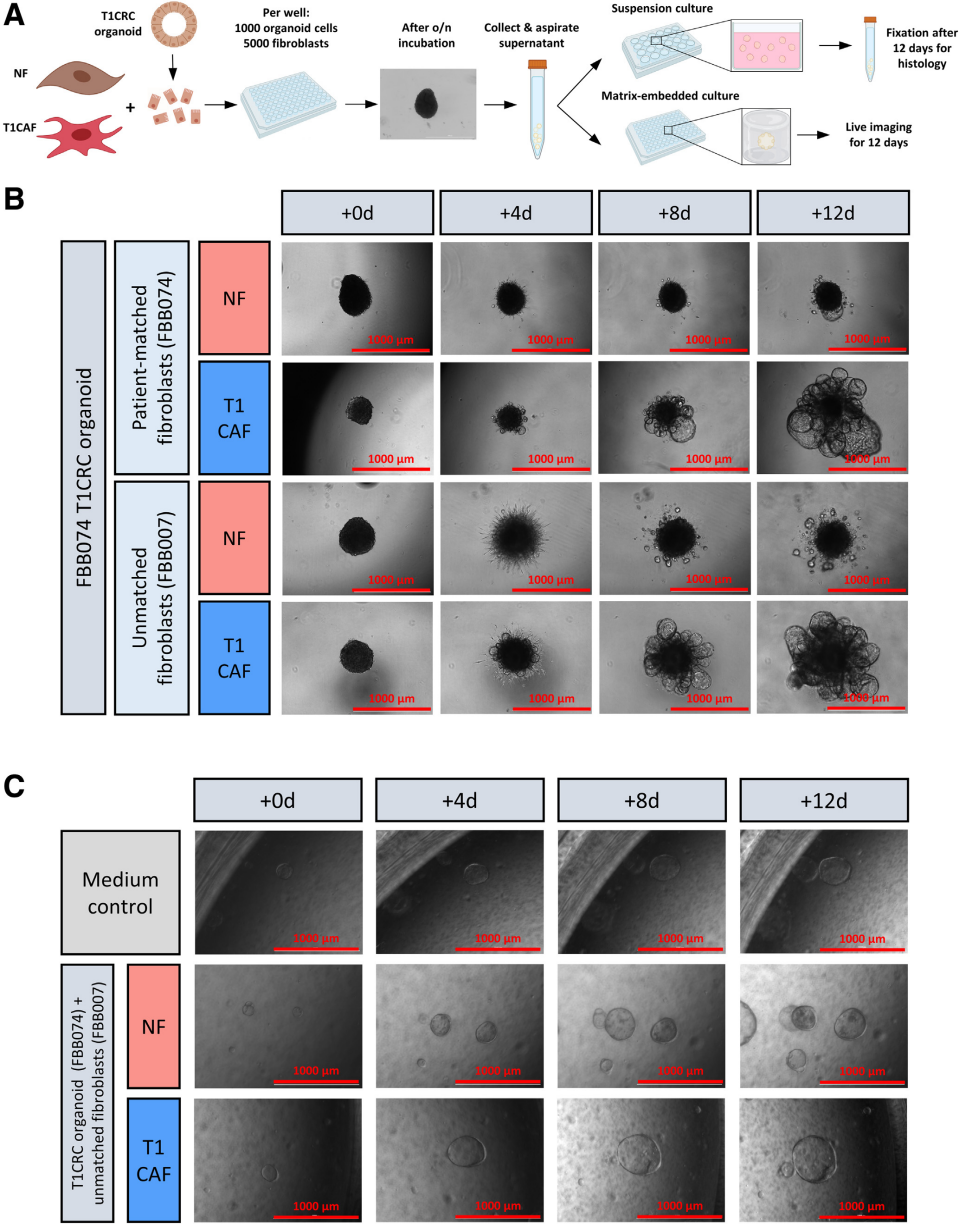
**Matrix-embedded and permeable membrane-separated co-cultures of primary NF-T1CAF pairs and T1CRC organoids.** (*A*) Workflow for organoid–fibroblast co-culture experiments. (*B*) Live imaging of Matrigel-embedded co-cultures of primary T1CRC organoids and (un)matched NF-T1CAF pairs. (*C*) Live imaging of permeable membrane-separated co-cultures of primary NF-T1CAFs with T1CRC organoids. Representative data are shown from 3 independent experiments (except for panel *C* and the co-culture with patient-matched organoids and fibroblasts: 2 independent experiments), each with 3 biological replicates all showing the same phenotype within each condition. o/n, overnight.

**Figure 3:**
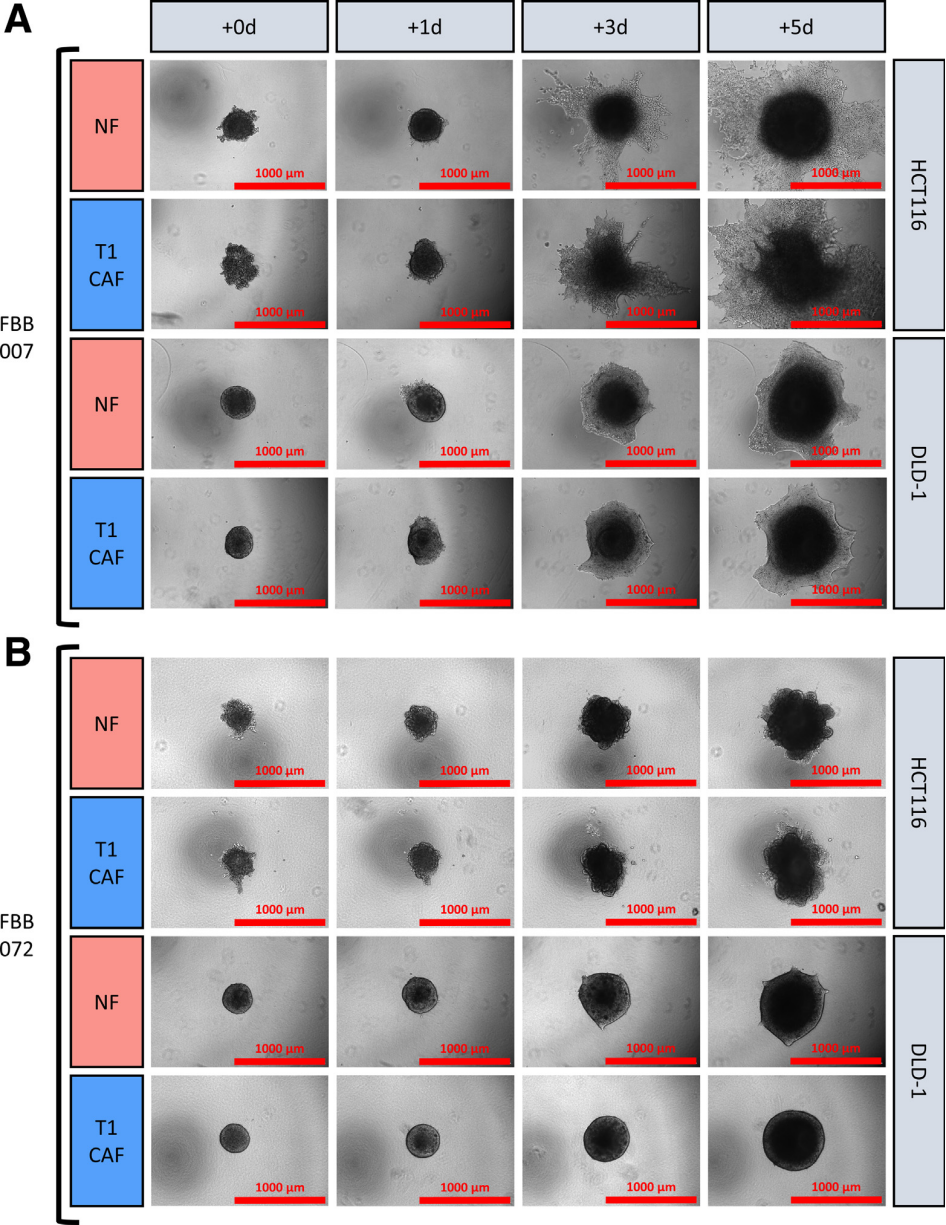
**3D-spheroid co-cultures of NF-T1CAF pairs and CRC cell lines.** Collagen-embedded 3D spheroid co-cultures of 2 CRC cell lines (HCT116 and DLD-1) with NF-T1CAF pairs from (*A*) patient FBB007 and (*B*) patient FBB072. Representative data are shown from 3 independent experiments, each with 3 biological replicates all showing the same phenotype within each condition.

**Figure 4:**
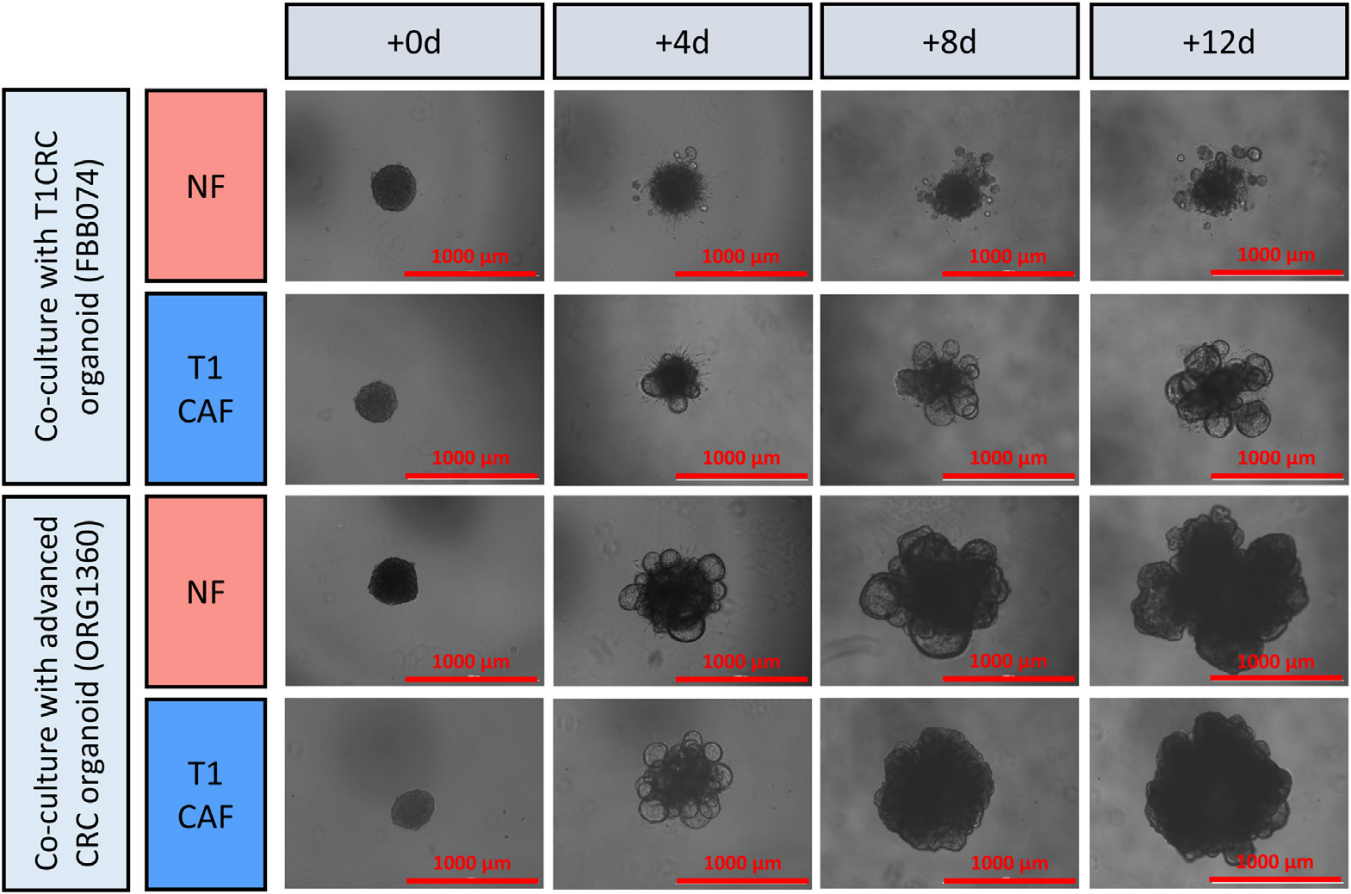
**Matrigel-embedded co-cultures of NF-T1CAF pairs and advanced CRC organoids.** Representative data are shown from 2 independent experiments, each with 3 biological replicates all showing the same phenotype within each condition.

To evaluate how T1CAFs and NFs affect tumor cell behavior, we established a novel 3D co-culture system of primary fibroblasts and T1CRC organoids. In our multicellular model, fibroblasts and organoid cells assembled into 3D aggregates, which then were embedded in a matrix for live imaging or cultured in suspension for histologic evaluation (Figure 2*A*). Using this model, we discovered that T1CAFs markedly promoted expansion of autologous (ie, both fibroblasts and organoids originating from the same patient) T1CRC organoids into growth factor–reduced Matrigel (Corning), which is composed primarily of 4 major basement membrane ECM proteins (ie, laminin, collagen type IV, entactin, and perlecan)^14^ (Figure 2*B*). Importantly, matched NFs did not induce this phenotype. Similar results were observed when using an allogeneic NF-T1CAF pair (Figure 2*B*). The observed differences in T1CRC behavior appeared to be dependent on direct cell–cell contact and not on paracrine signaling because T1CAFs and NFs did not differentially affect the phenotype of T1CRC organoids when separated with a permeable membrane (Figure 2*C*). Interestingly, T1CAFs and NFs also did not differentially affect cancer cell behavior when co-cultured with cell lines (Figure 3) or primary organoids (Figure 4) derived from more advanced CRCs (ie, >T1). This suggests a synchronization of both cancer cells and CAFs at the initial stages of CRC development.

**Figure 5:**
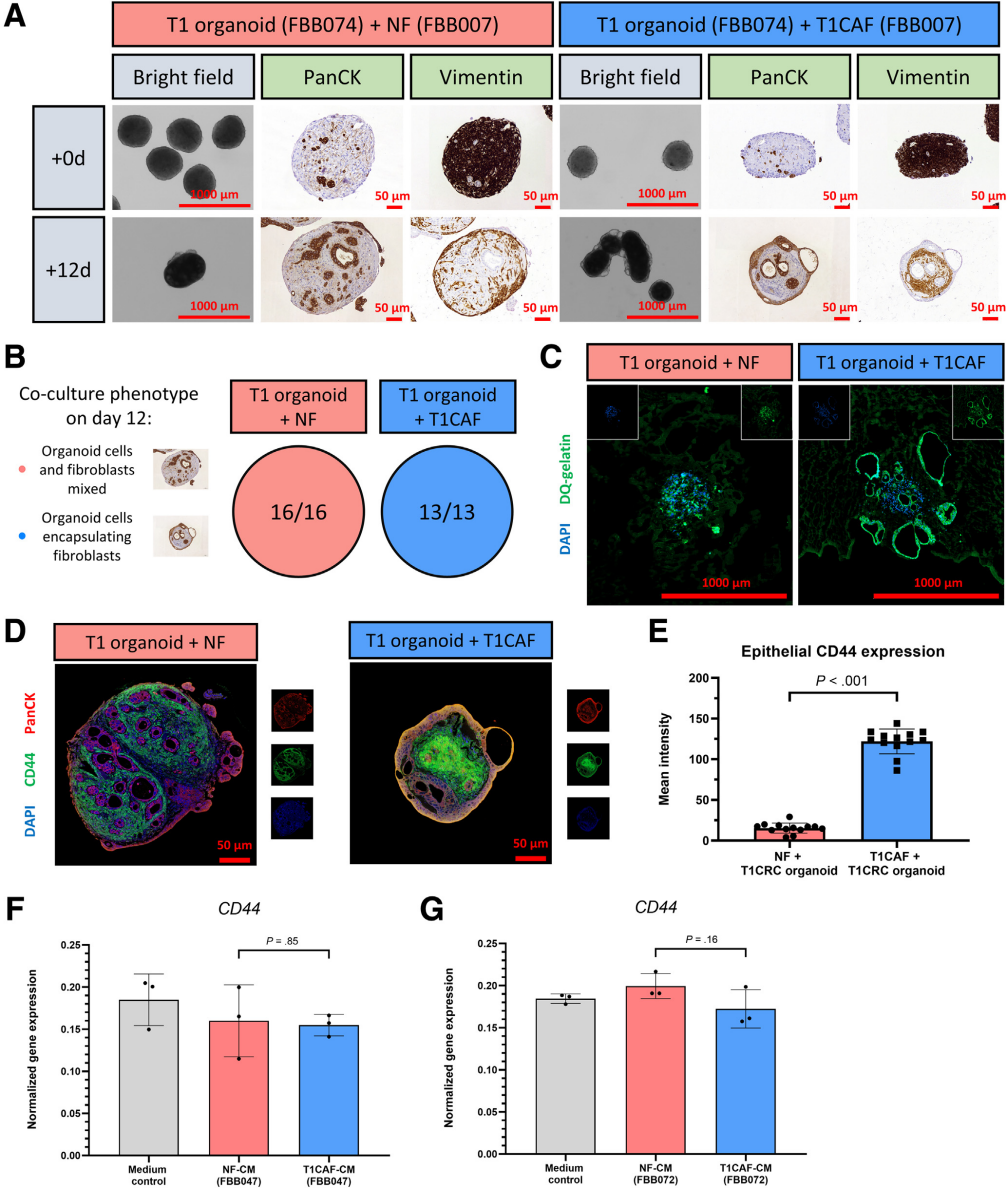
**T1CAFs promote Matrigel invasion of T1CRC organoids.** (*A*) Representative images of suspension co-cultures (FBB074 T1CRC organoid with unmatched NF-T1CAFs; fixed after 0 and 12 days) stained for a fibroblast (vimentin) and an epithelial (pancytokeratin) cell marker. (*B*) Quantification of organoid–fibroblast co-culture phenotypes. (*C*) In situ zymography of proteolytic activity with dye-quenced gelatin as substrate on cryosections of Matrigel-embedded co-cultures (FBB074 T1CRC organoid with unmatched NF-T1CAFs, frozen after 12 days; biological replicates all showing the same phenotype within each condition). (*D*) Immunostaining of suspension co-cultures (FBB074 T1CRC organoid with unmatched NF-T1CAFs; fixed after 12 days) for CD44 (green), pancytokeratin (PanCK) (red), and DAPI (blue). (*E*) Immunostaining quantification of epithelial CD44 expression (n = 16 NF-T1CRC organoid and n = 13 T1CAF–T1CRC organoid co-cultures). Gene expression levels of *CD44* in T1CRC organoids (FBB074) stimulated with conditioned medium of NF-T1CAF pairs from (*F*) patient FBB047 and (*G*) patient FBB072. Quantitative data are expressed as means ± SD and compared using the Student *t* test. CM, conditioned medium; DQ, dye-quenced.

**Figure 6:**
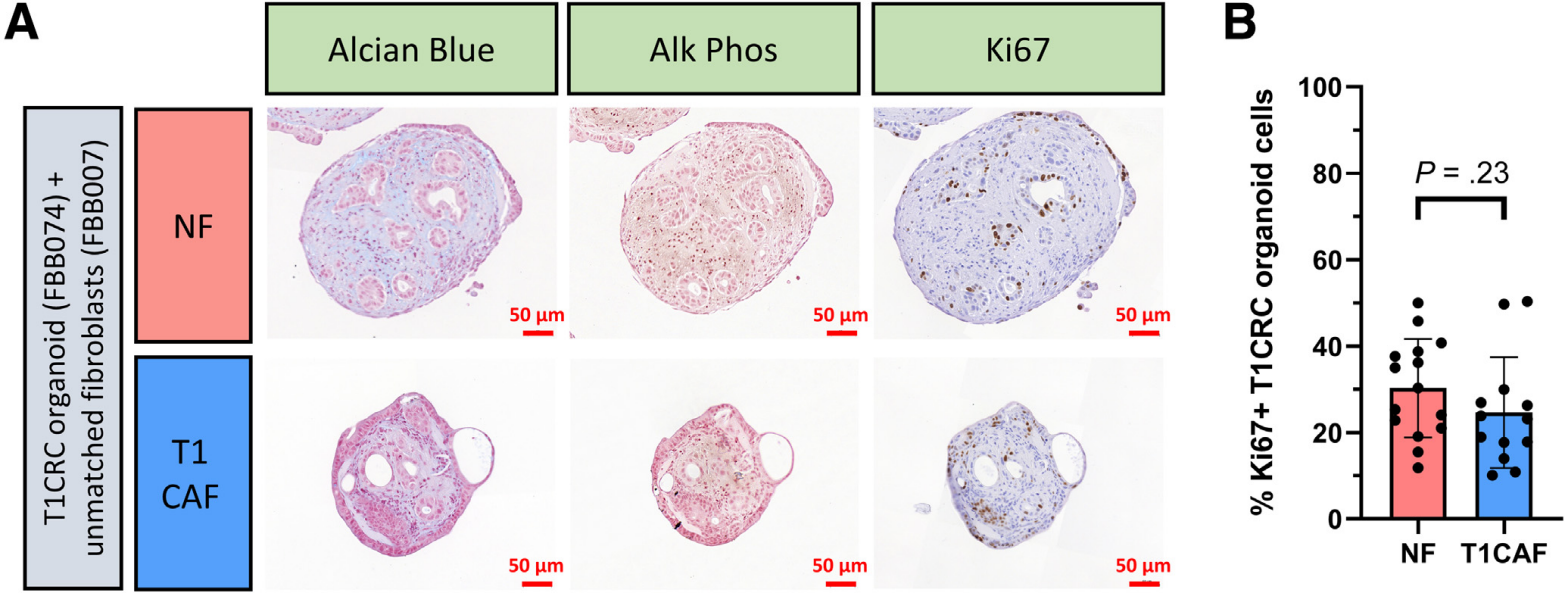
**T1CAFs do not promote proliferation or differentiation of T1CRC organoids.** (*A*) Suspension co-cultures of primary T1CRC organoids and unmatched NF-T1CAFs, stained for differentiation markers (Alcian blue, alkaline phosphatase [Alk Phos]; in both co-culture conditions none of the epithelial cells stained positive for these differentiation markers) and a proliferation marker (Ki67). (*B*) Quantification of the amount of Ki67-positive organoid cells in the co-cultures (n = 15 NF-T1CRC organoid and n = 13 T1CAF–T1CRC organoid co-cultures). All data are expressed as means ± SD and compared using the Student *t* test.

Histologic analysis revealed that NF and T1CAF–organoid co-cultures differed considerably in terms of cellular organization. At baseline, co-culture aggregates consisted of single or small clusters of cancer cells encapsulated by fibroblasts (Figure 5*A*). After 12 days of suspension culture, NFs still were encapsulating the organoid structures, whereas T1CAFs had re-organized into the core of the co-culture, with the cancer cells located on the outside (Figure 5*A* and *B*). To analyze whether or not T1CAFs induced invasion of T1CRC organoids into the matrix, in situ zymography on cryostat sections of Matrigel-embedded co-cultures was performed. This revealed high proteolytic activity at the outer rim of the T1CAF–organoid co-cultures (Figure 5*C*), suggestive of T1CAF-induced matrix invasion by the T1CRC organoids. In addition, T1CAF–organoid co-cultures showed increased epithelial expression levels of CD44 (Figure 5*D* and *E*), a transmembrane glycoprotein that is highly involved in tumor progression,^15^, ^16^, ^17^ and in particular in CRC invasion through basement membrane proteins.^18^, ^19^, ^20^ Strikingly, overall expression levels of *CD44* were not significantly different between monocultures of T1CRC organoids exposed to T1CAF and NF-conditioned medium (Figure 5*F* and *G*), thereby further emphasizing the dependency of T1CAF-induced organoid invasion on direct cell–cell contact. Lastly, the T1CAF-induced increase in matrix invasion by T1CRC organoids seemed not attributable to alterations in organoid proliferation or differentiation because epithelial expression levels of the proliferation marker Ki67 (Figure 6) and 2 differentiation markers (Figure 6*A*) did not differ between NF and T1CAF–organoid co-cultures. Together, these results show that T1CAFs can promote T1CRC invasion through basement membrane proteins via direct contact interactions with cancer cells.

### T1CAFs Promote Collagen Remodeling and Invasion of T1CRC

**Figure 7:**
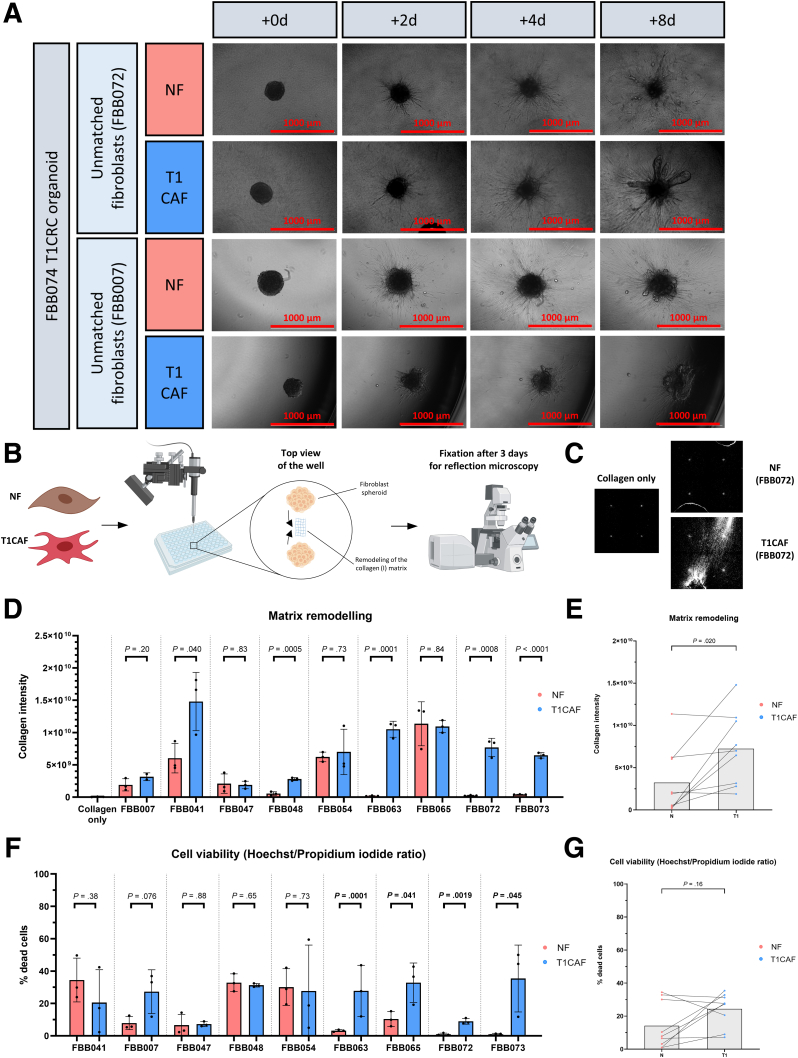
**T1CAFs promote collagen invasion of T1CRC organoids and collagen remodeling.** (*A*) Live imaging of collagen-embedded co-cultures of primary T1CRC organoids and organoid-unmatched NF-T1CAF pairs. (*B*) Workflow of matrix remodeling experiments with NF-T1CAF pairs. (*C*) Example reflection microscopy images of the collagen matrix (in between the 2 fibroblast spheroids). (*D* and *E*) Quantification of matrix remodeling by NFs and T1CAFs (n = 9 pairs). (*F* and *G*) Viability of NF-T1CAF pairs in matrix remodeling assays (n = 9 pairs). (*D* and *F*) Quantitative data are expressed as means ± SD and compared using the Student *t* test. (*E* and *G*) Matching NF-T1CAF pairs are connected with each other and compared using the Wilcoxon signed-rank test. Representative data are shown from 2 independent experiments, each with 3 biological replicates all showing the same phenotype within each condition.

Because T1CRCs have invaded not only the basement membrane but also the underlying submucosa, we next investigated whether T1CAFs also affect T1CRC invasion into a matrix of collagen type I, the main component of the intestinal submucosa.^21^ Using the earlier-mentioned multicellular model (Figure 2*A*), we found that T1CAFs markedly promoted invasion of T1CRC organoids into a collagen I matrix compared with matched NFs (Figure 7*A*) (patient ID number: FBB072). However, co-culture with a NF-T1CAF pair derived from another patient (Figure 7*A*) (FBB007) did not show this difference. Because both NFs and T1CAFs escaped from the co-culture aggregate into the collagen gel in the first 4 days (ie, before any differences in organoid invasion became apparent), we reasoned that the earlier-described observations (Figure 7*A*) might be explained by a difference in fibroblast-mediated matrix remodeling, which is instrumental for tumor progression.^22^, ^23^, ^24^ We therefore investigated whether there were any differences in the ECM remodeling capacity of NFs and T1CAFs. To this end, 9 NF-T1CAF pairs (including the 2 pairs that were used in the earlier-described co-culture experiments) were subjected to matrix remodeling assays. Two identical fibroblast spheroids were injected into a collagen type I gel in close proximity to each other, and matrix remodeling between the spheroids was visualized after 3 days using reflection microscopy (Figure 7*B* and *C*). Quantification of the reflection signal, which is indicative of collagen remodeling, revealed that T1CAFs showed significantly higher levels of ECM remodeling than matched NFs (Figure 7*D* and *E*). In particular, the T1CAFs that promoted collagen invasion (Figure 7*A*) **(**FBB072) also showed much more ECM remodeling than their normal counterparts, whereas the T1CAFs that did not promote invasion (Figure 7*A*) (FBB007) had comparable ECM remodeling capacity compared with matched NFs (Figure 7*D*). The observed differences could not be attributed to a decreased viability of NFs (Figure 7*F* and *G*), indicating that the enhanced ECM remodeling capacity is an intrinsic feature of T1CAFs. In summary, our findings indicate that T1CAFs can promote collagen invasion of T1CRC via increased collagen remodeling.

### ECM-Related Genes Are Differentially Expressed in T1CAFs

**Figure 8:**
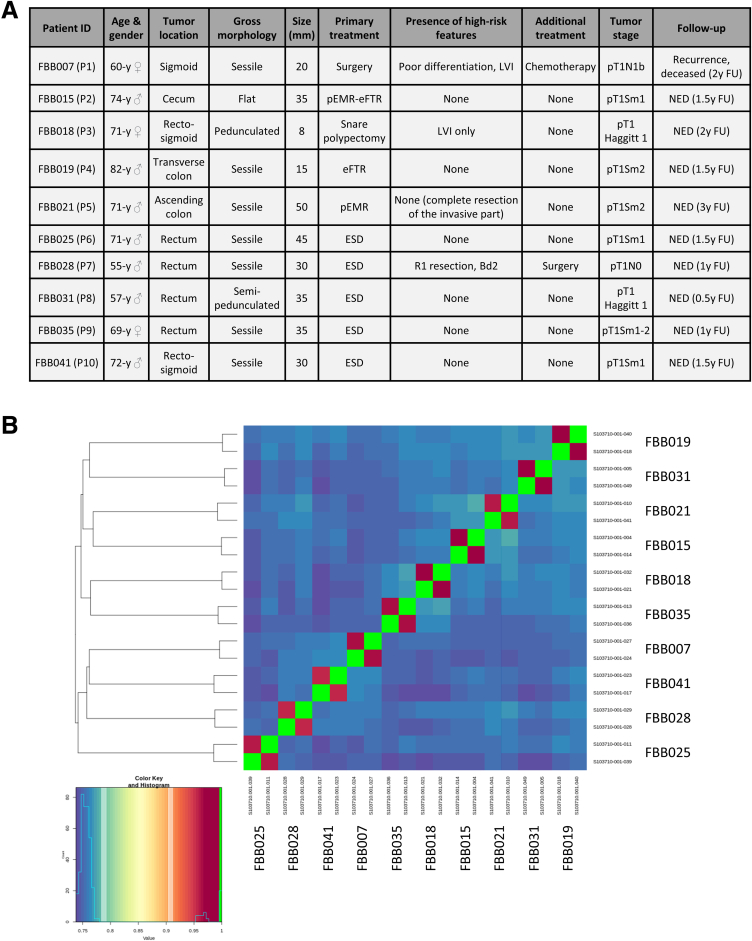
**Clinical characteristics of the patients in the mRNA sequencing cohort.** (*A*) Clinical characteristics of the patients in the sequencing cohort (n = 10 pairs). (*B*) Single-nucleotide polymorphism analysis to confirm that each NF-T1CAF pair originated from the same patient. Bd2, grade 2 tumor budding; eFTR, endoscopic full-thickness resection; ESD, endoscopic submucosal dissection; FU, follow-up; LVI, lymphovascular invasion; NED, no evidence of disease; pEMR, piecemeal endoscopic mucosal resection.

**Figure 9:**
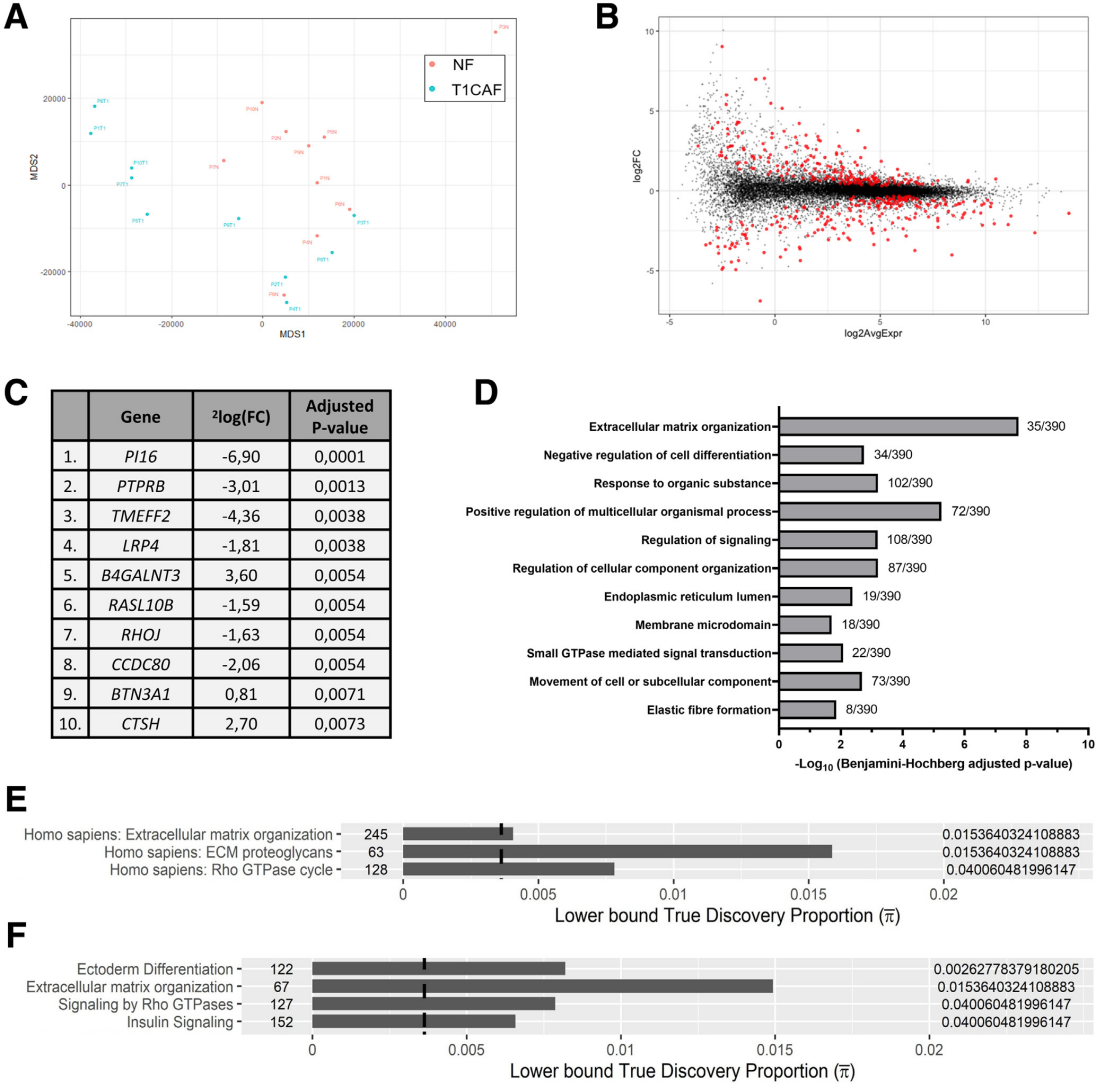
**mRNA sequencing of primary NF-T1CAF pairs.** (*A*) Principal component analysis of 10 matched NF-T1CAF pairs. (*B*) Volcano plot of differentially expressed genes between T1CAFs and NFs (statistically significant genes in red). (*C*) Top 10 most significant differentially expressed genes between T1CAFs and NFs. (*D*) Gene set enrichment analysis using DAVID update 2021. (*E*) Gene set enrichment analysis using rSEA, Reactome database. (*F*) Gene set enrichment analysis using rSEA, WikiList database. FC, fold change; GTPase, guanosine triphosphatase.

**Figure 10:**
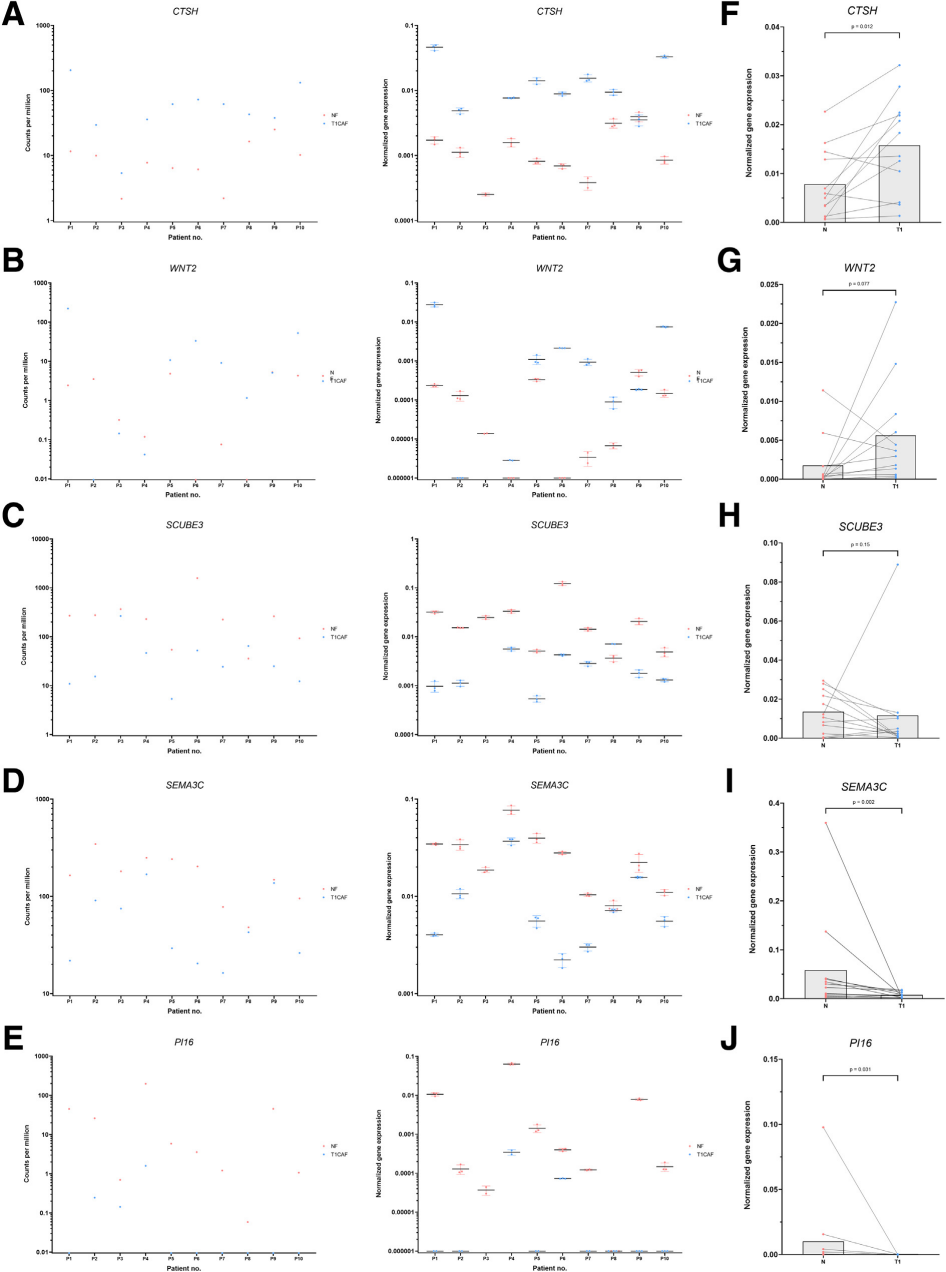
**Validation of differentially expressed genes in T1CAFs.** (*A*) Expression levels of (*A*) *CTSH*, (*B*) *WNT2*, (*C*) *SCUBE3*, (*D*) *SEMA3C*, and (*E*) *PI16* measured by mRNA sequencing (*left*) and qPCR (*right*). Expression levels of (*F*) *CTSH*, (*G*) *WNT2*, (*H*) *SCUBE3*, (*I*) *SEMA3C*, and (*J*) *PI16* in an independent cohort of T1CAFs and matched NFs (n = 12 pairs). FBB018 T1CAF (P3 from the sequencing cohort) could not be analyzed because of insufficient material. Matching NF-T1CAF pairs are connected with each other and compared using the Wilcoxon signed-rank test.

To identify factors underlying the phenotypic differences between T1CAFs and NFs, we performed messenger RNA (mRNA) sequencing of 10 matched NF-T1CAF pairs. The clinical characteristics of these 10 patients are shown in Figure 8*A*. Single-nucleotide polymorphism analysis confirmed that each pair of NFs and T1CAFs originated from the same patient (Figure 8*B*). Principal component analysis revealed that the NFs tended to cluster away from the T1CAFs (Figure 9*A*). We identified 404 differentially expressed genes between T1CAFs and NFs (q < 0.05) (Figure 9*B* and *C*, Supplementary Table 1). As expected, T1CAFs showed significantly lower expression levels of *PI16* (log_2_ fold change, -6.9; q = 0.001) (Figure 9*C*), a universal fibroblast marker that has been shown to be down-regulated upon fibroblast activation.^25^ Moreover, gene set enrichment analysis with 2 different algorithms showed that ECM-related ontology terms in particular were enriched significantly in T1CAFs (Figure 9*D–F*, Supplementary Tables 2–7). ECM-related differentially expressed genes included several collagens (*COL1A1*, *COL3A1*, *COL5A1*, and *COL23A1*) and *CTSH* (encoding cathepsin H, a cysteine protease that has been related to ECM remodeling^26^). We also found enrichment for other tumor progression-related processes such as cell migration and Wnt signaling (Supplementary Table 5) (examples of differentially expressed genes: *SCUBE3*, *SEMA3C*, *WNT2*). Next, we confirmed the differential expression of selected targets (*CTSH*, *SCUBE3*, *SEMA3C*, *WNT2*, and *PI16*) by qPCR on the same fibroblast pairs (Figure 10*A–E*). For 3 of these 5 genes (*CTSH*, *SCUBE3*, and *PI16*), differential expression also was validated in an independent cohort of 12 NF-T1CAF pairs (Figure 10*F–K*), thereby further confirming our findings. Taken together, T1CAFs are transcriptionally different from patient-matched NFs and mainly show differential expression of ECM-related genes.

### T1CAFs Show Stage-Specific Differential Expression of Multiple Genes

**Figure 11:**
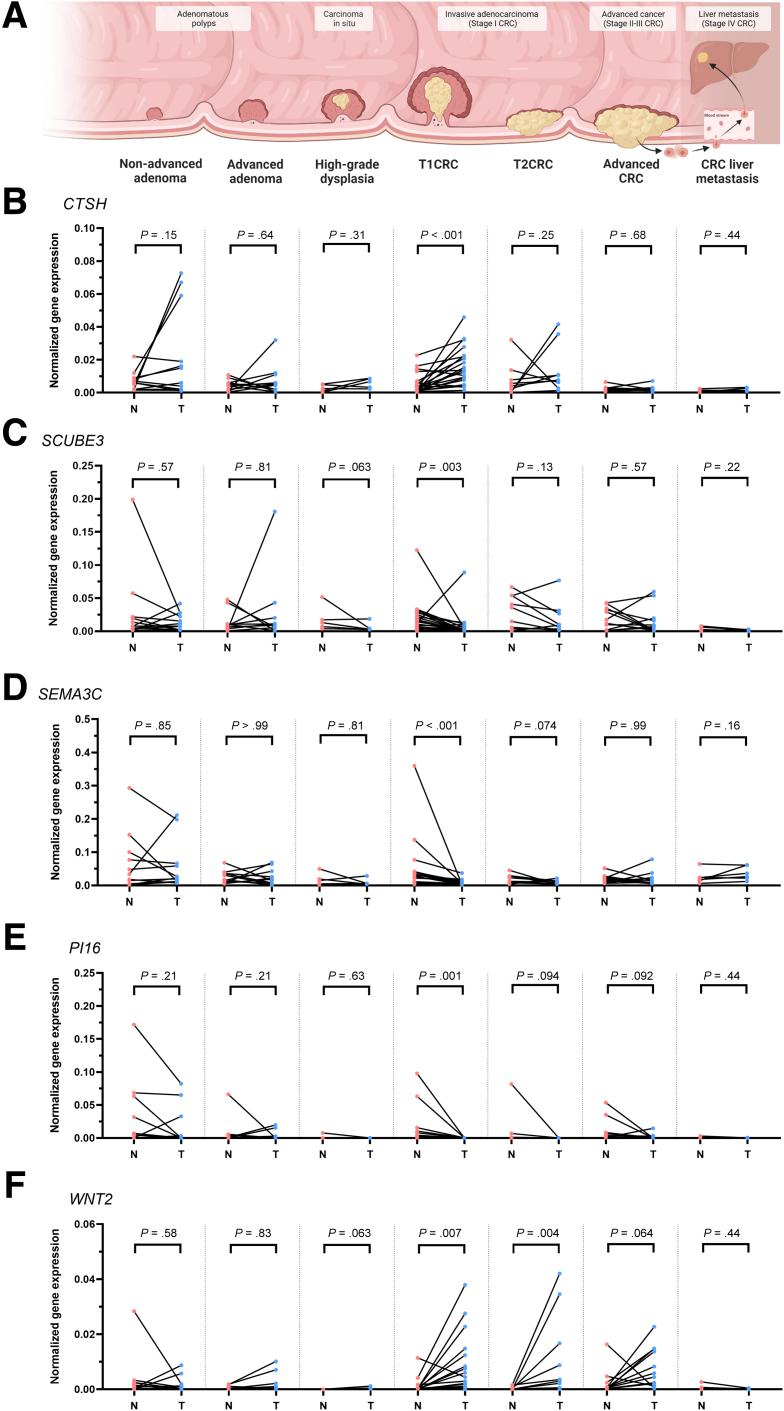
**Expression of T1CAF differentially expressed genes in tumor fibroblasts isolated from other CRC stages.** (*A*) Tumor stages of the adenoma–carcinoma sequence from which normal and tumor fibroblasts were isolated (nonadvanced adenoma cohort, n = 12 pairs; advanced adenoma cohort, n = 11 pairs; carcinoma in situ [ie, high-grade dysplasia] cohort, n = 5 pairs; T1CRC cohort, n = 21 pairs; T2CRC cohort, n = 9 pairs; advanced CRC cohort, n = 12 pairs; CRC liver metastasis cohort, n = 6 pairs). Expression levels of (*B*) *CTSH* (up-regulated in T1CAF vs NF), (*C*) *SCUBE3* (down-regulated in T1CAF vs NF), (*D*) *SEMA3C* (down-regulated in T1CAF vs NF), (*E*) *PI16* (down-regulated in T1CAF), and (*F*) *WNT2* (up-regulated in T1CAF vs NF). Matching NF-T1CAF pairs are connected with each other and compared using the Wilcoxon signed-rank test.

To examine whether or not differential expression of the earlier-described genes by T1CAFs is specific for the T1 stage, we performed extensive gene expression analyses of the 5 validated targets (*CTSH*, *SCUBE3*, *SEMA3C*, *WNT2*, and *PI16*). For these analyses, we took advantage of a large in-house biobank of 55 primary patient-matched, normal-tumor fibroblast pairs, covering all the different stages of the adenoma–carcinoma sequence (Figure 11*A*). These stages included nonadvanced adenomas (12 pairs), advanced adenomas (11 pairs), intramucosal carcinomas (5 pairs), stage I CRCs invading into the muscularis propria (T2 stage; 9 pairs), stages II–III CRCs (12 pairs), and stage IV CRCs (liver metastases; 6 pairs). Normal-tumor fibroblast pairs were isolated from endoscopic biopsy specimens or surgical resection material, and were processed in a similar way to the NF-T1CAF pairs (Figure 1*A*) to maximize the comparability of the samples. Strikingly, qPCR analysis revealed that *CTSH* was only differentially expressed in T1CAFs, but not in tumor fibroblasts from earlier or later CRC stages (Figure 11*B*). Likewise, *SCUBE3* and *SEMA3C* also showed expression patterns that appeared to be specific for early CRC stages (Figure 11*C* and *D*). Some targets (*PI16*, *WNT2*) not only showed differential expression in T1CAFs, but also in CAFs from later tumor stages (Figure 11*E* and *F*). Indeed, *WNT2* up-regulation and loss of *PI16* expression already have been reported in CAFs from advanced cancers.^25^^,^^27^^,^^28^ In short, T1CAFs show stage-specific differential expression of multiple targets (*CTSH*, *SCUBE3*, and *SEMA3C*) that were not recapitulated in fibroblasts from earlier or late-stage CRC.

### Enhanced Collagen Remodeling by T1CAFs Depends on Up-Regulation of Cathepsin H

**Figure 12:**
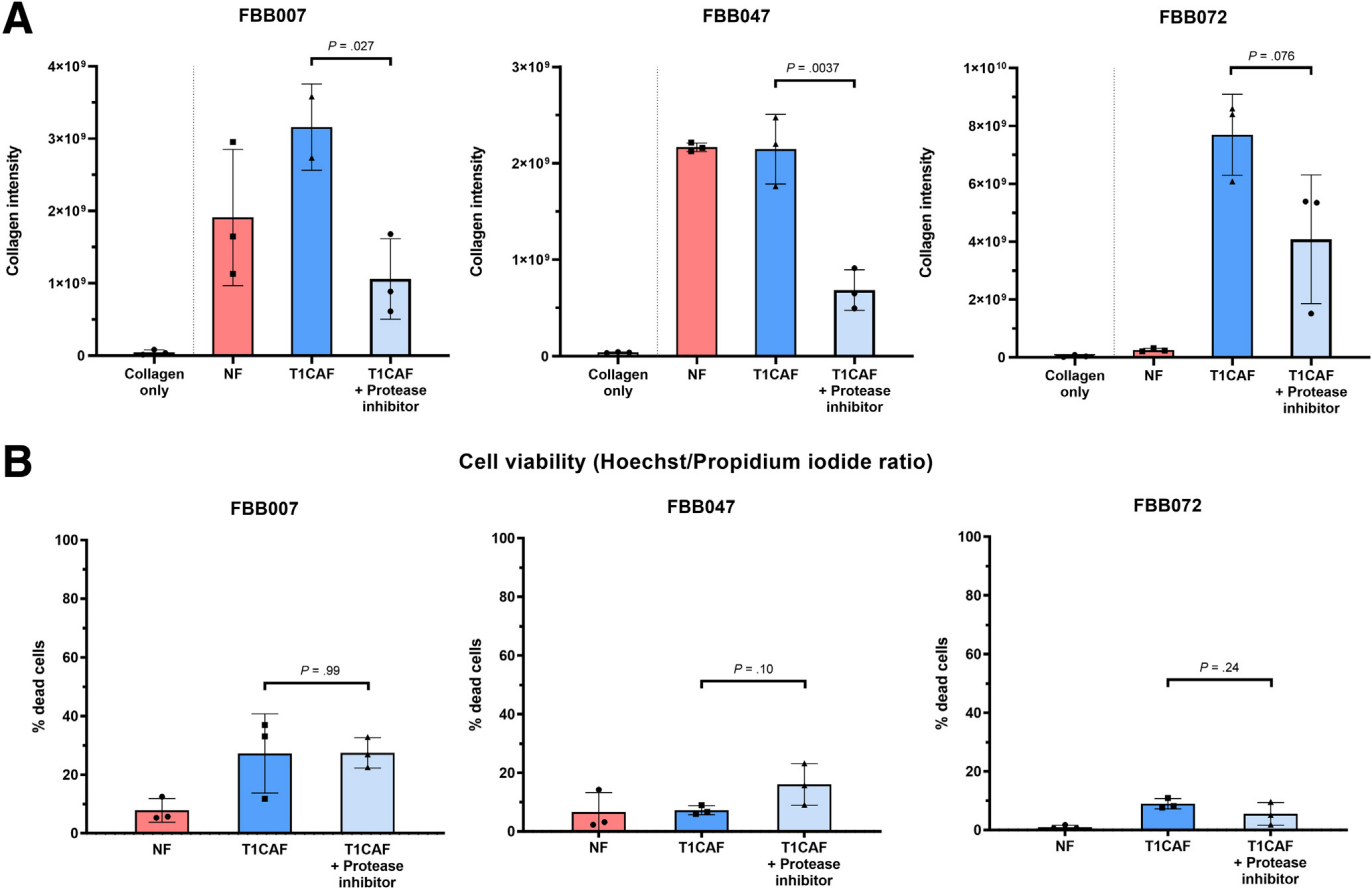
**Increased matrix remodeling by T1CAFs is protease dependent.** (*A*) Influence of protease inhibitors on matrix remodeling by T1CAFs (n = 3). (*B*) Cell viability of protease-treated T1CAFs in matrix remodeling assays (n = 3). Quantitative data are expressed as means ± SD and compared using the Student *t* test. Representative data are shown from 2 independent experiments.

**Figure 13:**
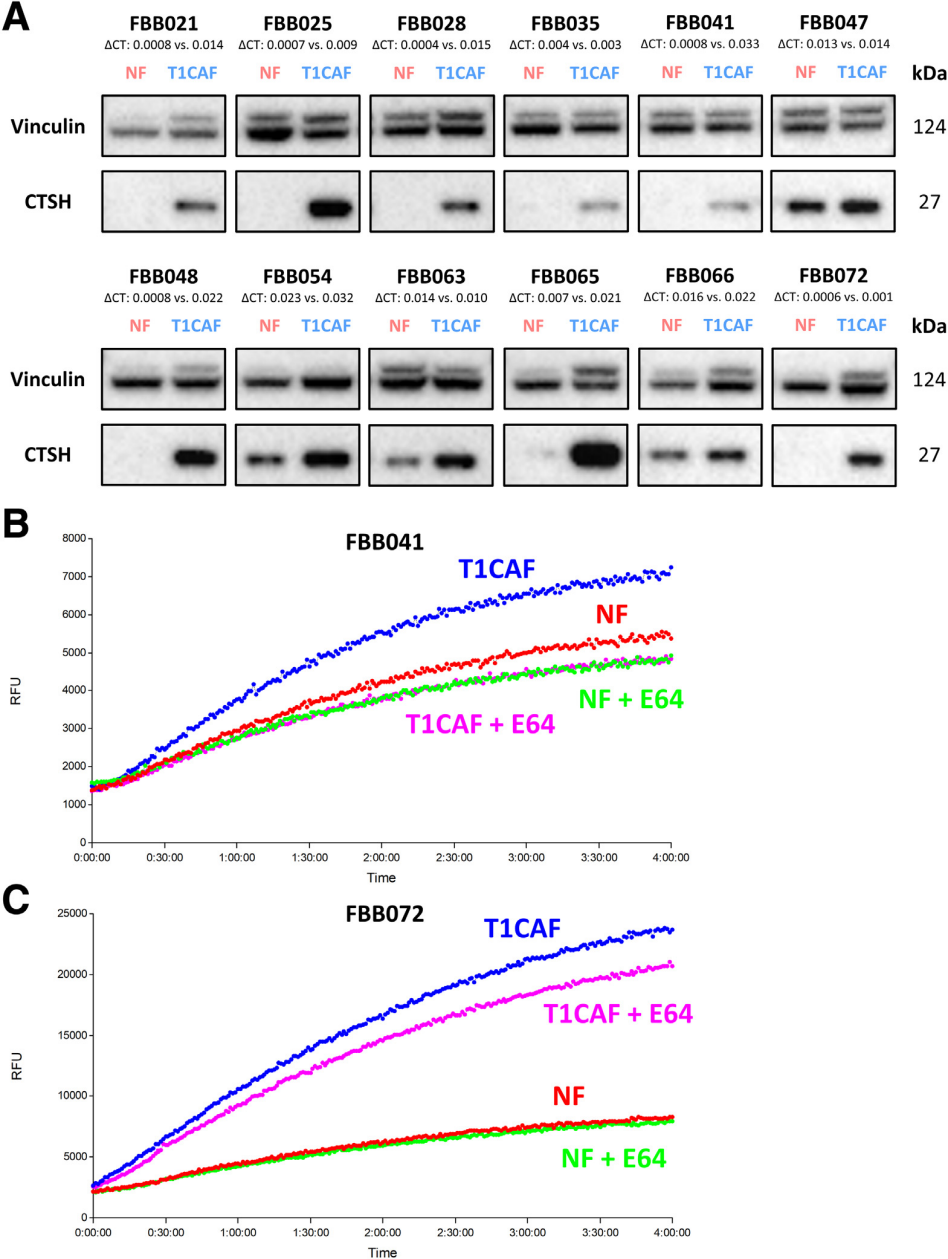
**Protein validation of cathepsin H up-regulation in T1CAFs.** (*A*) Western blot of cathepsin H expression in 12 NF-T1CAF pairs, with corresponding delta cycle threshold values from the qPCR analysis. Cathepsin H activity assays with conditioned medium of NF-T1CAF pairs, with and without the addition of the cysteine protease inhibitor E64, from (*B*) patient FBB041 and (*C*) patient FBB072. RFU: relative fluorescence units.

**Figure 14:**
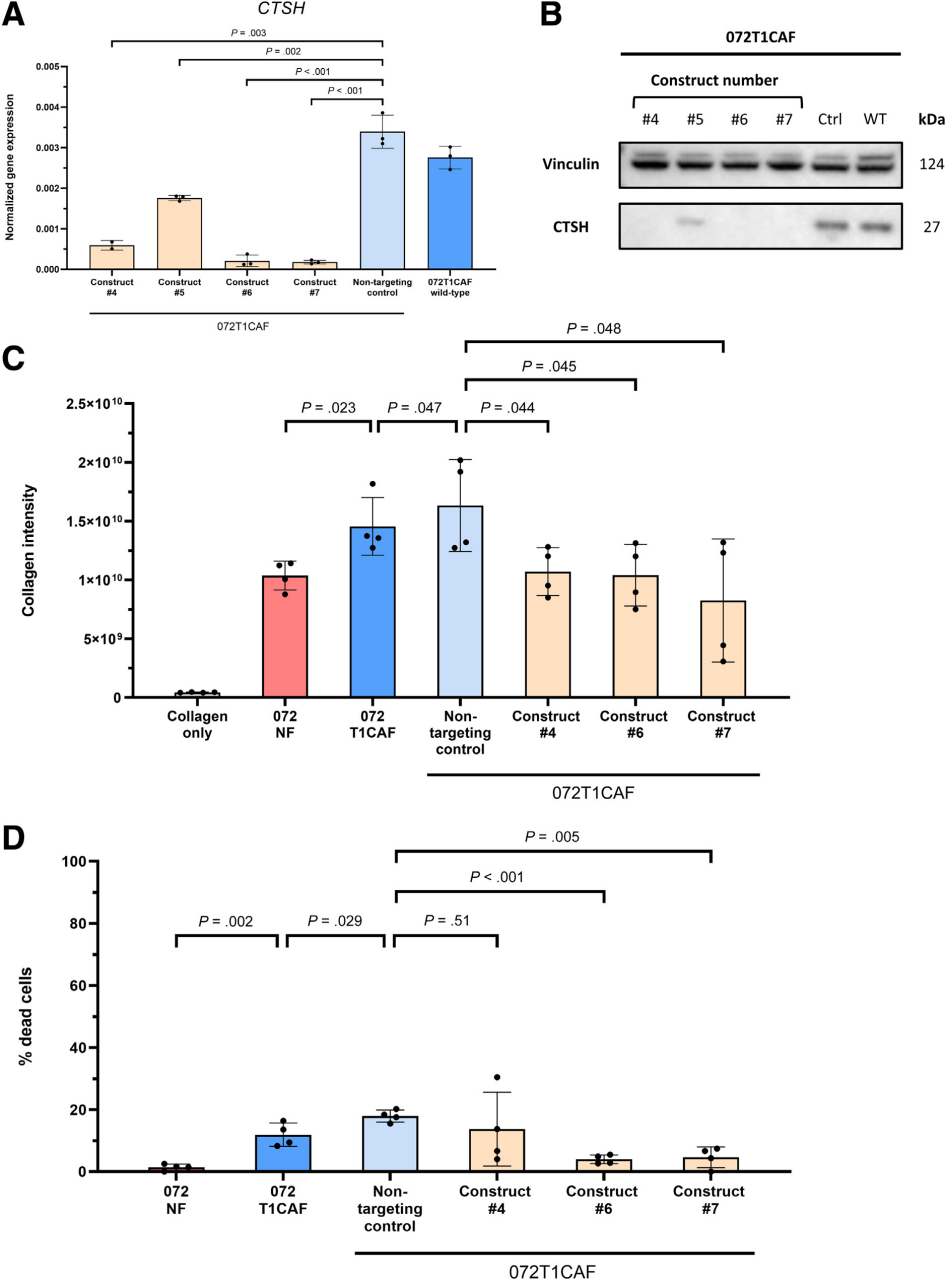
**Increased matrix remodeling by T1CAFs depends on up-regulation of cathepsin H.** (*A*) mRNA expression levels of CTSH in wild-type FBB072 T1CAF, vector control, and short hairpin RNA (shRNA)-mediated knockdown lines. (*B*) Western blot of cathepsin H expression in wild-type FBB072 T1CAF, vector control, and shRNA-mediated knockdown lines. Construct #5 was not included in the matrix remodeling experiments because of insufficient knockdown. (*C*) Influence of *CTSH* knockdown on matrix remodeling by T1CAFs (n = 4). (*D*) Cell viability of protease-treated T1CAFs in matrix remodeling assays (n = 4). Quantitative data are expressed as means ± SD and compared using the Student *t* test. Representative data are shown from 2 independent experiments.

Intrigued by the central role of T1CAFs in matrix invasion and their differential expression of ECM-related genes, we next focused on identifying the mechanisms underlying the enhanced matrix remodeling capacity of T1CAFs. First, using the earlier-mentioned remodeling assay (Figure 7*B*), we found that collagen remodeling by T1CAFs was dependent on protease activity because the addition of a broad-spectrum protease inhibitor resulted in significantly lower levels of remodeling in 3 independent T1CAFs (Figure 12*A* and *B*). Given the stage-specific up-regulation of the cysteine-type protease cathepsin H in T1CAFs (Figures 9*C* and 11*B*), we tested whether T1CAF-induced collagen remodeling was cathepsin H–dependent or not. We first confirmed the up-regulation of cathepsin H in primary T1CAFs compared with matched NFs using Western blot (Figure 13*A*). Cathepsin H activity assays also revealed increased activity of secreted cathepsin H in T1CAF-conditioned medium compared with their normal counterparts (Figure 13*B* and *C*). We next performed short hairpin RNA–mediated *CTSH* knockdown in primary T1CAFs from patient FBB072 because these T1CAFs markedly promoted collagen invasion of T1CRC (Figure 7*A*) and showed significantly higher levels of ECM remodeling than its normal counterpart (Figure 7*D*). After confirming successful knockdown on RNA and protein levels (Figure 14*A* and *B*), we subjected these fibroblasts to remodeling assays and found a significant decrease in collagen remodeling to levels of the matched NFs upon *CTSH* knockdown (Figure 14*C*). Again, this decrease could not be attributed to a decreased viability of the transduced T1CAFs (Figure 14*D*). In brief, these data show that the increase in collagen remodeling by T1CAFs depends on up-regulation of cathepsin H.

### Cathepsin H–Positive T1CAFs Are Abundant in Primary T1CRC Sections

**Figure 15:**
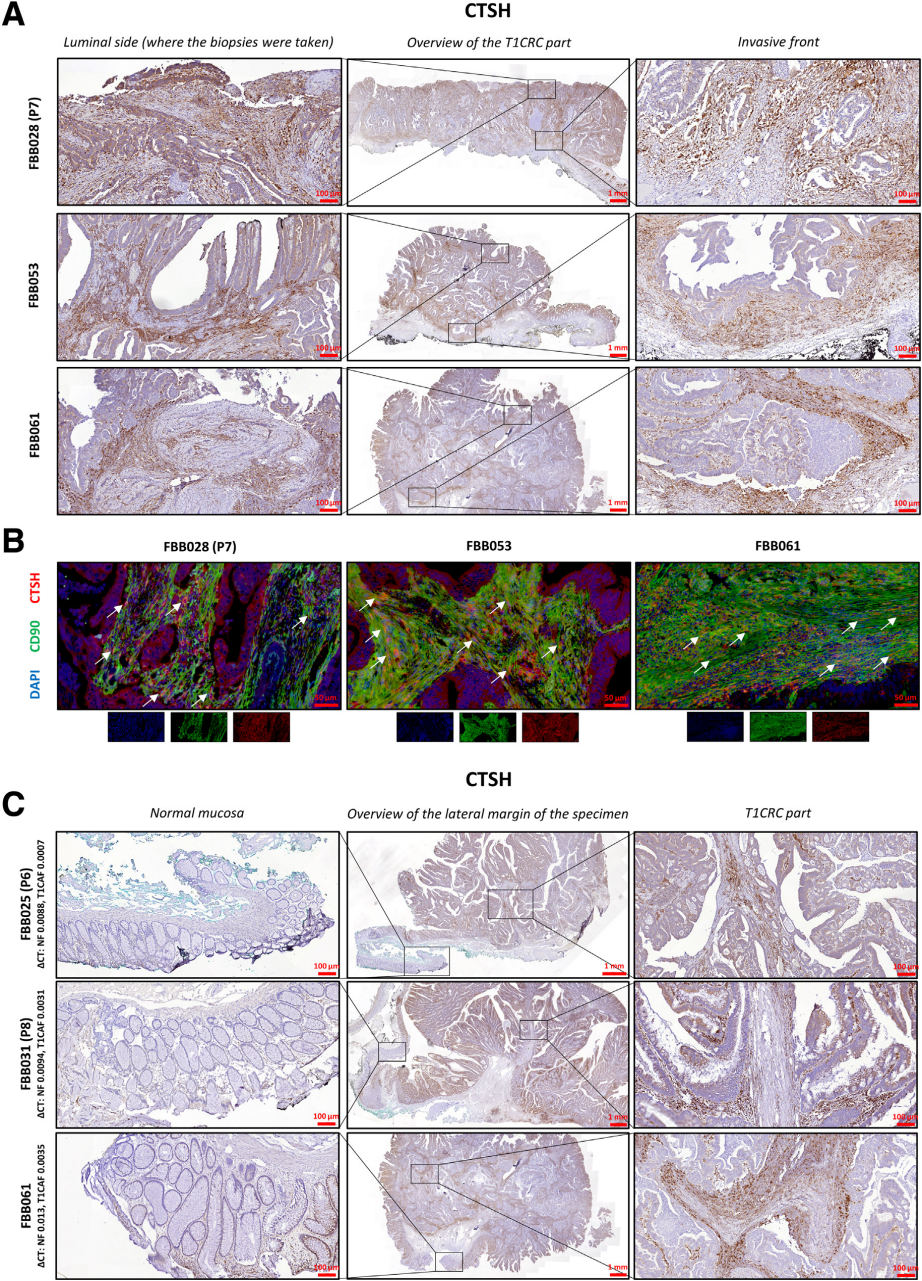
**Cathepsin H expression on primary T1CRC sections.** (*A*) Representative immunohistochemistry of primary T1CRC sections (T1CRC part) stained for cathepsin H. (*B*) Immunostaining of T1CRC sections for cathepsin H (green), CD90 (red), and DAPI (blue). (*C*) Representative immunohistochemistry of primary T1CRC sections (lateral margin of the resection specimen) for cathepsin H. ΔCT, delta cycle threshold.

Having identified cathepsin H as a driver of T1CAF-induced matrix remodeling, we next analyzed T1CAF-specific cathepsin H expression on primary T1CRC tissue sections. Slides of 22 endoscopically resected T1CRCs, which included cases from both the sequencing and validation cohorts, were stained for cathepsin H. Immunohistochemistry revealed high expression levels of cathepsin H in stroma-rich regions in the invasive part of the tumor, and in particular in fibroblast-like, spindle-shaped cells (Figure 15*A*). T1CAF-specific expression of cathepsin H was further confirmed by immunofluorescent double-staining of T1CRC sections for cathepsin H and the CAF marker CD90^6^ (Figure 15*B*). We also found that CAFs in the tumor stroma showed higher expression levels of cathepsin H compared with stromal cells in adjacent normal mucosa (Figure 15*C*). Finally, we observed that cathepsin H expression was present not only in superficial T1CAFs (ie, where the biopsy specimens were taken), but also in T1CAFs located in deeper tumor areas and at the invasive front (Figure 15*A*). This substantiates the generalizability of our findings on biopsy-isolated T1CAFs to deeper tumor parts, as well as indicating that the local interplay between T1CAFs, tumor cells, and ECM (as established in our in vitro experiments) may be instrumental for early CRC progression in vivo. In summary, we confirm the relative abundance of cathepsin H–positive T1CAFs on primary T1CRC sections.

## Discussion

Given the vast increase in the number of T1CRCs as a result of population-based screening and the major challenges in clinical management of these tumors, it has become crucial to obtain a better understanding of early stage tumor biology. In this study, we show that already in the earliest stage of invasive CRCs, T1CAFs are key determinants of cancer cell invasion. This study addresses the biology and functions of CAFs specifically at the initial stage of CRC development.

The importance of stage-specific investigation of CAFs is emphasized by our finding that multiple genes are exclusively differentially expressed in CAFs from early stage CRCs. Historically, CAFs have been studied extensively in advanced cancers, mainly because of the ease of obtaining large amounts of material from these tumors. However, recent work has highlighted the context-dependent heterogeneity of CAF populations,^6^^,^^7^^,^^10^^,^^29^ which hampers the generalizability of CAF studies. To illustrate this, novel spatial multiomic approaches have unveiled the gradual changes in fibroblasts that occur during cancer development.^12^^,^^30^^,^^31^ Although these studies only captured a small portion of the spectrum of cancer stages, their findings warrant rethinking the roles and functions that CAFs can play at different disease stages. Our study substantiates this point by providing direct empiric evidence for the existence of interstage heterogeneity of human CAFs along the entire continuum of CRC progression.

Studying T1CAF biology requires a different approach than previous CAF studies. This inherently is accompanied by several challenges, such as the very limited availability of T1CRC tissue and (cultured) cell materials for extensive experiments or experimental repeats with the same cell line. In addition, preclinical models also were lacking for T1CRCs that are representative of tumor biology in that particular stage. To tackle the latter issue, we almost exclusively used primary materials for experiments, as well as novel multicellular modeling techniques developed by our group^32^ to study the influence of T1CAFs on early tumor progression. Our findings underline the biological relevance of CAFs already at a very early stage in CRC development, because only changing the fibroblast part of the co-culture from NFs to T1CAFs was sufficient to drastically enhance matrix invasion of the same T1CRC organoid. Remarkably, we did not observe such phenotypic differences when co-culturing the same NF-T1CAF pairs with tumor cells derived from more advanced CRCs. This suggests that stage-specific investigation of CAFs also requires stage-specific, near-patient models that can recapitulate the context in which the CAFs reside in vivo.

Our transcriptomic profiling also indicates that T1CAFs are phenotypically different from their matched normal counterparts. Whole-transcriptome analysis revealed differential expression of several targets in T1CAFs, which have been related to tumor progression, such as *SCUBE3*,^33^, ^34^, ^35^ *SEMA3C*,^36^, ^37^, ^38^ and *WNT2*.^39^ The latter in particular has been studied extensively in the context of CAFs and CRC. For instance, Kramer et al^28^ showed that CAFs in advanced CRCs also display up-regulation of *WNT2*, and subsequent reports have highlighted the involvement of CAF-secreted Wnt2 in CRC migration and invasion,^27^^,^^28^ angiogenesis,^40^ and immunomodulation.^41^ Our data suggest that *WNT2* up-regulation in CAFs occurs very early in CRC development because T1CAFs already show increased expression of *WNT2* compared with matched NFs. Given that many recent studies have highlighted a considerable heterogeneity in CAF subsets and phenotypes that can be found within CRCs,^6^^,^^7^^,^^10^^,^^29^^,^^42^^,^^43^ it remains to be elucidated whether all T1CAFs or only a subpopulation of T1CAFs show differential expression of Wnt2 and other tumor-progression–related targets. Because direct ex vivo single-cell profiling of T1CAF subsets on endoscopic T1CRC biopsy specimens remains rather challenging because of the scarcity of the tissue, we propose multiomic-based strategies on residual (ie, after completing histologic evaluation for subsequent clinical decision making) whole T1CRC sections as more suitable alternatives for in-depth characterization of T1CAF subpopulations.

An important finding of our study was that T1CAFs importantly contribute to cancer cell invasion in several ways. For instance, they can induce invasion into a matrix of basement membrane proteins via direct cell–cell interactions. In line with this, our transcriptomic analyses revealed differential expression of multiple cell adhesion molecules in T1CAFs that have been associated with cancer cell invasion, such as *DSG2*,^44^, ^45^, ^46^ *CDH2*,^47^, ^48^, ^49^ *CDH10*,^50^ *PCDH9*,^51^^,^^52^ and *ITGB2*.^53^^,^^54^ Another way T1CAFs can facilitate cancer cell invasion is by actively remodeling the matrix into which the cancer cells invade. Our data show that T1CAFs show increased collagen remodeling capacity as well as differential expression of ECM-related genes compared with matched NFs. It is well known that ECM remodeling by CAFs contributes to tumor progression,^22^, ^23^, ^24^ with cancer cells leveraging the remodeled ECM for invasion and metastasis.^55^, ^56^, ^57^ A wide array of proteolytic enzymes can contribute to CAF-mediated matrix remodeling, including cysteine-type proteases.^26^^,^^58^, ^59^, ^60^, ^61^ Cathepsin H is one of the most poorly understood members of the cysteine proteases. It shows both aminopeptidase and endopeptidase activity,^61^^,^^62^ and often is suggested, together with the other cathepsins, to be involved in matrix organization.^26^^,^^58^ Here, we showed that the enhanced matrix remodeling capacity of T1CAFs at least partly depends on up-regulation of cathepsin H. The presence of cathepsin H–expressing T1CAFs also was confirmed at the invasive front of primary T1CRCs, suggesting that cathepsin H–dependent matrix remodeling by T1CAFs contributes to cancer cell invasion.

Intriguingly, consistent up-regulation of cathepsin H was observed only in T1CAFs and not in tumor fibroblasts from any other CRC stage, indicative of a T1-specific mechanism of matrix remodeling. Possible explanations might include the local interplay with epithelial cells in T1CRC or the presence of certain intestinal submucosa–specific matrix components or characteristics, which only can be processed through up-regulation of cathepsin H in CAFs. Because the ECM substrates of cathepsin H largely are unknown, it is yet not clear whether cathepsin H shows direct proteolytic activity on the matrix or contributes to a cascade that results in the activation of other proteases. To further elucidate on this question, it might be valuable to perform extensive substrate profiling of cathepsin H as well as in-depth biomechanical and biomolecular characterization of the submucosa in health and disease.

The clinical implications of our findings can be sought mainly in providing promising leads for markers that can predict aggressive disease of T1CRC patients. These markers could contribute to reducing the large number of unnecessary additional surgeries after organ-preserving local resection. Our previous study on T1CRCs did not detect a significant association between clinical outcome and total stromal content, as determined by the tumor–stroma ratio.^63^ However, given that this parameter only accounts for the total amount but not the composition of the stroma, as well as the biological relevance of T1CAFs in early CRC progression, we think that T1CAF-derived signatures may contain more predictive power, as also hinted at in a small Japanese cohort study.^64^ It would be of interest to investigate whether the amount of cathepsin H–expressing T1CAFs could be of prognostic value for T1CRC patients. Another candidate marker that particularly holds promising potential are T1CAFs expressing Wnt2. This is because Wnt2 expression levels in CAFs specifically already have been shown to be associated significantly with lymph node metastasis and prognosis of advanced CRCs,^27^^,^^28^ as well as esophageal squamous cell carcinomas.^65^ Adequately powered epidemiologic studies are required to evaluate the predictive value of T1CAF-derived biomarkers in T1CRCs.

In conclusion, our study shows that already in the earliest stage of CRC, CAFs are important determinants of tumor progression. T1CAFs already have undergone phenotypic changes that make them entirely different from normal fibroblasts, but they also show certain stage-specific traits that are not recapitulated in tumor fibroblasts from other CRC stages. We show that T1CAFs are able to markedly increase matrix invasion of T1CRC as well as promote ECM remodeling in a cathepsin H–dependent manner. These findings provide fertile ground for the improvement of risk stratification and clinical management of T1CRC patients.

## Methods

### Human Ethics and Data Availability

The study was approved by the Medical Ethical Committee of Leiden University Medical Center (reference numbers B20.039 and B22.036) and conforms to the latest version of the Declaration of Helsinki (2013). Written informed consent for study participation was provided by all included T1CRC patients. Patients or the public were not involved in the design, conduct, reporting, or dissemination plans of the research. All human tissues were handled according to the Dutch Code of Conduct for Responsible Use of Human Tissues (Federa-COREON, 2011). RNA sequencing data of the 10 NF-T1CAF pairs can be found under the GEO accession number GSE200660. Other data supporting the study findings are available from the corresponding author upon reasonable request.

### Primary NF-T1CAF Isolation and Culture

Primary patient-matched T1CAFs and NFs were isolated from fresh endoscopic biopsy specimens of T1CRC and normal tissue, respectively. During colonoscopy, 2–6 targeted biopsy specimens were taken from tumor regions with optical features of submucosal invasion (eg, Kudo Vi pit pattern or Sano IIIa vessel pattern^4^), and from normal adjacent mucosa 5–10 cm away from the tumor. Patient-matched biopsy specimens were taken during the same procedure and within the same intestinal segment to maximize comparability of the samples. All included T1CRC cases had to be confirmed histologically by an expert gastrointestinal pathologist (S.C.). T1CRC was defined as histologic tumor invasion through the muscularis mucosa and into, but not beyond, the submucosa. Biopsy specimens were collected in fibroblast medium (Dulbecco’s 123 modified Eagle medium [DMEM]/F12 GlutaMAX supplemented with 10% fetal calf serum [FCS], 50 μg/mL gentamycin, 2.5 μg/mL fungizone, 100 IU/mL penicillin, and 100 μg/mL streptomycin; all from Thermo Fisher Scientific, Waltham, MA) and digested with a 3:1 mix of collagenase type II (Thermo Fisher Scientific) and dispase II (Roche, Basel, Switzerland) for 2 hours at 37°C. After digestion, single-cell suspensions were plated in cell culture dishes (Greiner Bio-One, Kremsmünster, Austria) and cultured in fibroblast medium at 37°C and 5% CO_2_. Outgrowth of fibroblast-like cells was observed after 2–7 days. Media were changed weekly. Cells were cultured for up to 8 passages for experiments. All cells were tested routinely for mycoplasma contamination using PCR analysis.

### Primary Fibroblast Isolation and Culture

Primary patient-matched, normal-tumor fibroblast pairs of nonadvanced adenomas, advanced adenomas, intramucosal carcinomas, and T2CRCs were isolated from endoscopic biopsy specimens in a similar way as the NF-T1CAF pairs. Targeted tumor biopsy specimens were taken based on optical diagnosis (features of noninvasive polyps: eg, Kudo III–IV pit pattern^66^ and Sano II vessel pattern^67^; features of T2CRCs: eg, Kudo Vn pit pattern,^66^ Sano IIIb vessel pattern,^67^ and Borrmann type 2 or 3 tumor^68^), which subsequently was confirmed on histology. Advanced adenomas were defined as tubular adenomas >10 mm or adenomas with villous histology, and nonadvanced adenomas as tubular or tubulovillous adenomas ≤10 mm.^69^ Intramucosal carcinomas were defined as noninvasive (ie, not invading through the muscularis mucosae) tumors with high-grade dysplasia.^70^ T2CRC was defined as histologic tumor invasion into, but not beyond, the muscularis propria.^71^

Primary patient-matched, normal-tumor fibroblast pairs from stages II–IV CRCs (classified according to the American Joint Committee on Cancer system^71^) were isolated from non-necrotic parts of surgically resected tumors and adjacent normal mucosa (American Joint Committee on Cancer stages II–III CRCs), or from surgically resected CRC liver metastases and adjacent normal liver tissue (American Joint Committee on Cancer stage IV CRCs). Tissues were washed in phosphate-buffered saline (PBS), minced into small fragments, and then processed for fibroblast isolation in a similar way as the endoscopic biopsy specimens.

### Primary Organoid Isolation and Culture

Primary organoids were isolated from fresh endoscopic biopsy specimens of histologically confirmed T1CRC, and primary advanced CRC organoids from non-necrotic parts of surgically resected tumors. The biopsy specimens/tissue fragments were collected and digested to single-cell suspensions as described previously. Cell suspensions subsequently were plated in growth factor–reduced, phenol red–free Matrigel (Corning, Corning, NY) domes and overlaid with organoid medium. The medium for T1CRC organoids consisted of advanced DMEM/F12, 2 mmol/L L-glutamine, 100 IU/mL penicillin and 100 μg/mL streptomycin, 1× B27 supplement (all from Thermo Fisher Scientific), 1.25 mmol/L N-acetyl cysteine (Sigma, St Louis, MO), 10 mmol/L nicotinamide (Sigma), 50 nmol/L A83-01 (Tocris Bioscience, Bristol, UK), 10 μmol/L SB202190 (STEMCELL Technologies, Vancouver, Canada), 0.5 nmol/L Wnt-surrogate-Fc fusion protein (Utrecht Protein Express, Utrecht, The Netherlands), 100 ng/mL recombinant human epidermal growth factor (Peprotech, Cranbury, NJ), 100 ng/mL recombinant noggin (Peprotech), 100 ng/mL recombinant R-spondin 3 (Peprotech), and 100 μg/mL Primocin (Invivogen, San Diego, CA). The medium for advanced CRC organoids consisted of the earlier-described T1CRC organoid medium without the Wnt-surrogate-Fc fusion protein and SB202190, and with the addition of 10 nmol/L gastrin (Sigma). Media were changed every 2–3 days. Organoids were passaged using TrypLE Express Enzyme (Thermo Fisher Scientific), and 10 μmol/L ROCK inhibitor (Y-27632; Bio-Techne, Minneapolis, MN) was added to the organoids during the first 1–2 days after dissociation to prevent anoikis.

### CRC Cell Line Culture

The CRC cell lines HCT116 and DLD-1 were obtained from the American Type Culture Collection and were cultured in DMEM supplemented with 10% FCS, 100 IU/mL penicillin, and 100 μg/mL streptomycin (all from Thermo Fisher Scientific).

### 3D Organoid-Fibroblast Co-Cultures

Primary T1CRC organoids were trypsinized to single-cell suspensions, and NF-T1CAF suspensions were obtained from trypsin-detached adherent cultures. Organoids and fibroblasts were mixed in a 1:5 ratio in DMEM/F12 GlutaMAX supplemented with 10% FCS, 100 IU/mL penicillin, 100 μg/mL streptomycin (all from Thermo Fisher Scientific), 10 μmol/L ROCK inhibitor (Bio-Techne), and 1% Matrigel (Corning). The mixtures were plated in ultra-low-attachment, 96-well, round-bottom plates (Corning; n = 1000 organoids and n = 5000 fibroblasts per well), centrifuged (1200 rpm for 5 minutes), and incubated overnight at 37°C in 5% CO_2_. The next day, the formed aggregates were collected and the supernatant was removed. For matrix-embedded cultures, individual aggregates were taken up in 100% Matrigel or 1 mg/mL collagen type I (diluted in fibroblast medium; Ibidi, Gräfelfing, Germany) and plated in triplicate in flat-bottom, 96-well plates (Greiner) that were precoated with 50 μL of corresponding matrix. After polymerization of the matrix, 100 μL organoid medium (see *Primary Organoid Isolation and Culture* section) without A83-01 was added. For suspension cultures, 30–50 aggregates were taken up in organoid medium (also without A83-01) containing 1% Matrigel and plated in ultra-low-attachment, 6- or 24-well, flat-bottom plates (Corning). Media were changed every 2 days. Live imaging of suspension and matrix-embedded cultures was performed every 2 days using a Cytation 5 Cell Imaging Multi-Mode Reader (BioTek Instruments, Inc, Winooski, VT). After 12 days, the co-cultures were processed for histologic evaluation. Matrix-embedded cultures were scooped out from the 96-well plate using a small spoon. The whole gel (with the co-culture inside) was transferred to a small tin can with a cork plate on the bottom of the can. The can then was filled with Tissue-Tek O.C.T. compound (Sakura, Osaka, Japan), snap-frozen in melting isopentane, and stored at -80°C. Each co-culture of an experimental triplicate was frozen in a separate can. Suspension cultures were washed with PBS, fixed in 4% paraformaldehyde for 10 minutes, dehydrated using increasing concentrations of ethanol, cleared with xylene, and embedded in paraffin.

### 3D CRC Cell Line–Fibroblast Co-Cultures

The CRC cell lines HCT116 and DLD-1 were mixed together with NF-T1CAF pairs in a 1:1 ratio in CRC medium (see *CRC Cell Line Culture* section) and 0.24% methylcellulose. The mixtures were plated in 96-well, round-bottom plates (Corning; n = 2000 CRC cells and n = 2000 fibroblasts per well), centrifuged (1200 rpm for 5 minutes), and incubated overnight at 37°C in 5% CO_2_. The next day, the formed aggregates were collected and the supernatant was removed. Individual aggregates were taken up in 1 mg/mL collagen type I (diluted in CRC medium; Ibidi) and plated in triplicate in flat-bottom, 96-well plates (Greiner) that were precoated with 50 μL collagen type I. After polymerization of the collagen, 100 μL CRC medium was added. Live imaging of the co-cultures was performed daily using a Cytation 5 Cell Imaging Multi-Mode Reader (BioTek Instruments, Inc).

### Permeable Membrane-Separated Organoid–Fibroblast Co-Cultures

Primary T1CRC organoids were trypsinized to single-cell suspensions and plated in growth factor–reduced, phenol red–free Matrigel (Corning) domes that were placed in the upper compartment of 12-mm Transwell cell culture inserts (0.4-μm pore size; Corning). The organoids were overlaid with organoid medium, and 10 μmol/L ROCK inhibitor (Bio-Techne) was added during the first 1–2 days. Four days after organoid dissociation, NF-T1CAF suspensions were obtained from trypsin-detached adherent cultures and seeded in 12-well plates. The next day, the Transwell inserts with the organoid domes were transferred to the 12-well plates with the fibroblasts. Organoid medium without A83-01, recombinant human epidermal growth factor, noggin, R-spondin 3, and Wnt-surrogate-Fc fusion protein, was added to the upper and lower compartments of the Transwell inserts. A medium control (ie, no fibroblasts in the lower compartment) also was included. Live imaging of the organoids was performed every 2 days using a Cytation 5 Cell Imaging Multi-Mode Reader (BioTek Instruments, Inc).

### NF-T1CAF Conditioned Medium Experiments

For collection of NF and T1CAF-conditioned medium, fibroblasts were cultured in culture dishes to ≥75% confluency and then were kept in serum-free DMEM/F12 medium for 3–4 days. Thereafter, conditioned medium was harvested, centrifuged to remove cell debris, and stored at -20°C. Freshly thawed aliquots were used for all conditioned medium experiments. For gene expression analyses, confluent T1CRC organoids were stimulated in triplicate with T1CAF and NF-conditioned medium for 6 hours. A medium control (serum-free DMEM/F12) also was included in the stimulation experiment. T1CRC organoids then were washed with PBS, and Matrigel domes containing the organoids were depolymerized on ice using Cell Recovery Solution (Corning). After depolymerization, organoid suspensions were washed twice with cold PBS and centrifuged at 1200 rpm for 5 minutes to harvest RNA pellets.

### ECM Remodeling Assays

Single-cell suspensions of trypsin-detached NF-T1CAF pairs were filtered, centrifuged at 1000 rpm for 5 minutes, and resuspended in ∼30–60 μL PBS containing 2% polyvinylpyrrolidone (Sigma). Cell suspensions were injected in solidified 1.5 mg/mL collagen type I gels in black FLUOTRAC 200 flat-bottom, 96-well plates (VWR International, Radnor, PA), as described previously.^72^ To study collagen remodeling between fibroblast spheroids, fibroblasts were injected at an approximately 500-μm distance and incubated in DMEM/F12 GlutaMAX supplemented with 10% FCS, 100 IU/mL penicillin, and 100 μg/mL streptomycin until spheroid diameters reached approximately 200 μm. Fibroblast treatments included the addition of a broad-spectrum protease inhibitor (cOmplete tablets; Roche). Collagen remodeling was analyzed by reflection microscopy as described previously^73^ using a Nikon TE2000 confocal microscope (Nikon, Tokyo, Japan) with a 40× long distance water immersion objective by illuminating with a 561 laser coupled with a 561 blocking dichroic mirror for the detection and capture of the total reflection signal. Fibroblast cell viability was analyzed by incubating the cultures with Hoechst 33342 (Thermo Fisher) and propidium iodide, and determining the intensity of the propidium iodide signal for each nucleus identified by the Hoechst signal in a total of 10 Z-stacks.

### RNA Isolation and Real-Time qPCR

Total RNA was isolated using the Nucleospin RNA kit or RNA XS kit (both from Macherey-Nagel, Düren, Germany) according to the manufacturer’s instructions. Complementary DNA was synthesized from 0.25 to 1.0 μg RNA using the RevertAid First Strand Complementary DNA Synthesis Kit (Thermo Fisher). Real-time qPCR was performed with SYBR Green Master mix (Bio-Rad, Hercules, CA) using the CFX96 Touch Real-Time PCR Detection System (Bio-Rad). Expression values were normalized to β-actin expression. The sequences of all primers used can be found in Table 1.

**Table 1:** All Primer Sequences

Gene	Forward primer, 5’---3’	Reverse primer, 5’---3’
*CTSH*	TACCTTCGAGGTACTGGTCCCT	GGTGGAGAAAGTCCAGCAACTG
*WNT2*	AGGATGCCAGAGCCCTGATGAA	AGCCAGCATGTCCTGAGAGTAC
*PI16*	CTGGTGTGCAACTATGAGCCTC	GGCAAATCCTGAGCATCTTCCG
*SCUBE3*	CTCCAGGCAAAGAGGTCACAAG	TCCTTTCAGCCGCCGTTCCATT
*SEMA3C*	ACCCACTGACTCAATGCAGAGG	CAGCCACTTGATAGATGCCTGC
*ACTA2*	CCGGGAGAAAATGACTCAAA	GAAGGAATAGCCACGCTCAG
*VIM*	TGTCCAAATCGATGTGGATGTTTC	TTGTACCATTCTTCTGCCTCCTG
*FAP*	ATCTATGACCTTAGCAATGGAGAATTTGT	GTTTTGATAGACATATGCTAATTTACTCCCAAC
*CD31*	GCTGACCCTTCTGCTCTGTT	TGAGAGGTGGTGCTGACATC
*CD45*	AACAGTGGAGAAAGGACACA	TGTGTCCAGAAAGGCAAAGC
*KRT20*	CAGACACACGGTGAACTATGG	GATCAGCTTCCACTGTTAGACG
*ACTB*	GTTGTCGACGACGAGCG	GCACAGAGCCTCGCCTT

### Messenger RNA Sequencing and Transcriptomic Analyses

For messenger RNA (mRNA) sequencing, the total RNA of NF-T1CAF pairs from 10 different patients was isolated. The concentration and integrity of the RNA were measured using a BioAnalyzer (Agilent Technologies, Wilmington, DE). Samples with a RNA integrity number ≥8 were subjected to mRNA sequencing (RNA integrity number range of analyzed samples, 9.6–10).

RNA sequencing libraries were prepared from total RNA using the NEBNext Ultra II Directional RNA Library Prep Kit for Illumina (New England Biolabs, Ipswitch, MA) according to the manufacturer’s instructions. mRNA sequencing (15 million paired-end reads of 150 bp per sample) with poly(A) tail enrichment was performed by ServiceXS (GenomeScan, Leiden, The Netherlands) using the Illumina NovaSeq 6000 (San Diego, CA). FastQC was used for checking raw read quality.^74^ Adapter clipping was performed using Cutadapt (v2.4) with default settings. RNA sequencing read alignment was performed using STAR (v2.6.0c) on the GRCh38 human reference genome. The gene read quantification was performed using HTSeq-count (v0.9.1) with the -stranded=reverse setting. The gene annotation used for quantification was Ensembl version 94. Single-nucleotide polymorphism transcript analysis was performed as a quality control to confirm that all fibroblast pairs were matched correctly (ie, normal and tumor fibroblast pairs obtained from the same patient). Unsupervised principal component analysis was performed using the *stats* package v3.6.2. Differential gene expression was performed using *voom/limma* (v3.48.3)^75^ after removing low-expressed genes (read counts, < 5) and read count normalization (using the trimmed mean of M values (TMM) normalization method^76^).

Gene set enrichment analysis was performed using 2 different algorithms. The first method was the DAVID 2021 update^77^ (medium stringency criteria for grouping, gene set annotations: GO_BP_FAT, GO_CC_FAT, GO_MF_FAT, REACTOME_PATHWAY, and WIKIPATHWAYS), which allows for clustering of related gene annotations. The second method is rSEA v2.1.1 (gene annotation databases: GO_BP [biological processes; org.Hs.eg.db, Bioconductor v3.10], GO_CC [cellular components; org.Hs.eg.db, Bioconductor v3.10], GO_MF [molecular functions; org.Hs.eg.db, Bioconductor v3.10], Reactome [reactome.db, Bioconductor v3.10], and Wikipathways database [rWikiPathways, Bioconductor v3.10]), which does not rely on the unrealistic assumption of independence of features.^78^ *P* values of rSEA analyses were controlled for the family wise error rate; *P* values of all other sequencing analyses and gene set enrichment analyses were adjusted according to the Benjamini–Hochberg^79^ method to correct for multiple testing.

### DNA Isolation and Sequencing

DNA was isolated from organoid cell pellets and snap-frozen T1CRC biopsy specimens using the Nucleospin DNA RapidLyse kit (Macherey-Nagel) according to the manufacturer’s instructions. Next-generation sequencing was performed with the CHPv6 panel (Department of Pathology, Leiden University Medical Center, Leiden, The Netherlands), a customized AmpliSeq panel (Thermo Fisher Scientific) used for sequencing hotspot regions that frequently are mutated in human cancer genes. These genes included *ABL1*, *AKT1*, *ALK*, *APC*, *ARAF*, *ATM*, *BAP1*, *BRAF*, *BRCA2*, *CARD11*, *CCND1*, *CD79A*, *CD79B*, *CDH1*, *CDK4*, *CDKN2A*, *CIC*, *CRNKL1*, *CSF1R*, *CTNNB1*, *DDR2*, *DICER1*, *EGFR*, *EIF1AX*, *ERBB2*, *ERBB3*, *ERBB4*, *ERCC2*, *EZH2*, *FBXW7*, *FGFR1*, *FGFR2*, *FGFR3*, *FLT3*, *FOXL2*, *GNA11*, *GNAQ*, *GNAS*, *H3F3A*, *H3F3B*, *HNF1A*, *HRAS*, *IDH1*, *IDH2*, *JAK2*, *JAK3*, *KDR*, *KIT*, *KNSTRN*, *KRAS*, *MAP2K1*, *MAP2K2*, *MAP2K4*, *MAP3K1*, *MDM2*, *MED12*, *MET*, *MLH1*, *MPL*, *MUTYH*, *MYC*, *MYD88*, *NKX2-1*, *NOTCH1*, *NPM1*, *NRAS*, *NTRK1*, *PDGFRA*, *PDGFRB*, *PIK3CA*, *POLD1*, *POLE*, *PPP2R1A*, *PTEN*, *PTK2*, *PTPN11*, *RB1*, *RET*, *SMAD4*, *SMARCB1*, *SMO*, *SRC*, *STK11*, *TP53*, and *VHL*. Detected alterations classified as (likely) pathogenic (class 4 or 5, respectively) are reported.

### Gene Silencing in Primary T1CAFs

Lentiviral particles were generated using third-generation packaging vectors and human embryonic kidney 293 cells expressing a mutant version of the Simian vacuolating virus 40 large T antigen (HEK293T cells).^80^ Knockdown constructs for cathepsin H were acquired from the Mission TRC1 short hairpin RNA library (Sigma). Target sequences were 5’-ATGGATGTCTAAGCACCGTAA-3’, 5’-GATAAAGTAAACCATGCAGTA-3’, 5’-GACGCAAAGATCACCAGCCAT-3’, and 5’-GAGGAAGATAAACGCCCACAA-3’. Cells were selected and cultured with 1.5 μg/mL puromycin (Sigma).

### Western Blot

Fibroblast pellets were lysed in RIPA buffer with a broad-spectrum protease inhibitor (cOmplete tablets; Roche). The protein content was determined using the DC protein assay (Bio-Rad) according to the manufacturer’s instructions. Equal amounts of protein (20–30 μg; diluted in 4× sample buffer [125 mmol/L Tris/HCl, pH 6.8; 4% sodium dodecyl sulfate, 2% β-mercaptoethanol, 20% glycerol, 1 mg bromophenol blue]) were heated at 95°C for 5 minutes and separated with electrophoresis using precast polyacrylamide gradient gels (NuPAGE 4%-12%, Bis-Tris; Thermo Fisher Scientific) under reducing conditions. Proteins were transferred to polyvinylidene difluoride membranes (Merck, Readington, NJ). Nonspecific binding was blocked in 5% milk powder in Tris-buffered saline containing 0.05% Tween-20 (Merck). Blots were incubated overnight with mouse anti–cathepsin H and mouse antivinculin (both from Santa Cruz Biotechnology, Santa Cruz, CA). Detection was performed by horseradish peroxidase–conjugated goat anti-mouse antibodies (Agilent Technologies) and chemiluminescence (Lumi-Light Western Blot Substrate; Roche) was used to visualize the target proteins.

### Cathepsin H Activity Assays

Recombinant inactive human cathepsin H (Bio-Techne) was activated according to the manufacturer’s instructions. Approximately 200–300 μL conditioned medium of NF-T1CAF pairs was filtered and concentrated to 40–50 μL using Amicon Ultra-2 centrifugal filter units (Sigma). A total of 10 μL concentrated medium, with or without the addition of the irreversible cysteine protease inhibitor E-64 (working concentration, 10 μmol/L; Sigma), was loaded in a black FLUOTRAC 200 flat-bottom, 96-well plate (VWR International), supplemented by 40 μL assay buffer (50 mmol/L 2-morpholinoethanesulfonic acid monohydrate, pH 6.5; Sigma). Positive (100 ng recombinant cathepsin H) and negative controls (50 μL assay buffer) were included for each activity assay. After loading all samples, the cathepsin H substrate (50 μL of 200 μmol/L L-arginine-7-amido-4-methylcoumarin hydrochloride; Sigma) was added to the wells. Directly thereafter, the fluorescence-quenched substrate was measured at ex/em = 380/460 nm. Measurements were taken every minute for 4 hours using a Cytation 5 Cell Imaging Multi-Mode Reader (BioTek Instruments, Inc).

### Histochemical and Immunohistochemical Staining

For histochemical staining, formalin-fixed, paraffin-embedded tissues were sectioned in 4-μm sections. Mounted slides were deparaffinized, rehydrated, and stained for Alcian Blue (1% Alcian Blue; Merck; and 3% acetic acid solution) or alkaline phosphatase (NBT/BCIP Ready-to-Use Tablets; Sigma) for 30 minutes. Slides were counterstained with Neutral Red (Merck), rinsed in tap water, dehydrated, and mounted with Entellan (Merck).

For immunohistochemistry (IHC) or immunofluorescent (IF) staining, formalin-fixed, paraffin-embedded tissue sections 4-μm thick were deparaffinized. For IHC, additional blocking in 0.3% hydrogen peroxidase (Merck) in methanol was performed for 20 minutes. Next, for both IHC and IF, slides were rehydrated, and antigen retrieval was performed by boiling in 0.01 mol/L sodium citrate (pH 6.0) for 10 minutes. Slides then were washed and incubated with primary antibody diluted in 1% PBS/bovine serum albumin overnight at room temperature in a humidified box. Primary antibodies used in this study were mouse antipancytokeratin (Sigma), rabbit antivimentin (Cell Signaling Technology, Danvers, MA), rabbit anti–cathepsin H (Bio-Techne), mouse anti-CD44 (Abcam, Cambridge, UK), mouse anti-CD90 (Bio-Techne), and rabbit anti-Ki67 (Abcam). The next day, slides were washed and incubated with appropriate biotinylated secondary antibodies (Agilent Technologies) or anti-mouse Alexa 488 and anti-rabbit Alexa 568 (both from Thermo Fisher Scientific). For IF, slides then were mounted with ProLong Gold Antifade reagent with 4′,6-diamidino-2-phenylindole (DAPI) (Thermo Fisher Scientific). For IHC, slides were incubated with Vectastain complex (Vector Laboratories, Newark, CA) at room temperature for 30 minutes. Staining was visualized by incubating slides with Dako Liquid DAB+ Substrate Chromogen (Agilent Technologies) at room temperature for 10 minutes. Nuclei were counterstained with Mayer’s Hematoxylin (Merck). Finally, IHC slides were rinsed in tap water, dehydrated, and mounted with Entellan (Merck).

Slides were scanned with the Pannoramic 250 slide scanner (version 1.23; 3DHISTECH, Ltd, Budapest, Hungary). Images were obtained using Caseviewer (version 2.5; 3DHISTECH, Ltd) or using an Olympus BX51 Light Microscope (Olympus, Tokyo, Japan) equipped with an Olympus DP25 camera.

Quantification of epithelial CD44 expression in organoid–fibroblast co-cultures was performed using QuPath (version 0.3.1)^81^ by first segmenting nuclei using DAPI and expanding the cells by 20 pixels to capture the entirety of the cells. Next, cells were classified as epithelial cells or other cells by the presence or absence of epithelial marker (pancytokeratin), after which the expression levels of CD44 were determined in all epithelial cells and compared between samples.

### In Situ Zymography

Unfixed, frozen, Matrigel-embedded, organoid–fibroblast co-cultures were cut in 8-μm thick cryostat sections, and mounted slides were stored at -20°C. Proteolytic activity on the cryosections was evaluated using dye-quenced gelatin (Thermo Fisher Scientific) as a substrate.^82^ Dye-quenced gelatin was dissolved in a concentration of 1 mg/mL in water and then 1:10 diluted in 1% (w/v) low-gelling-temperature agarose (Sigma) in PBS containing 1 μg/mL DAPI (Sigma) to counterstain nuclei. The mixture was put on top of air-dried cryosections and covered with a coverslip. After gelling the agar at 4°C, the incubation was started at room temperature for 1 hour, followed by incubation in humidified cassettes at 37°C for 24 hours. Fluorescence of the quenched substrate was detected at ex/em = 469/525 nm. Images were taken using a Cytation 5 Cell Imaging Multi-Mode Reader (BioTek Instruments, Inc).

### Statistical Analyses

All statistical analyses were performed using GraphPad Prism v9.0.1 (GraphPad Software) or R v4.1.2. Continuous variables were compared using the Student *t* test (unpaired samples) or the Wilcoxon signed-rank test (paired samples). A *P* value less than .05 was considered statistically significant.
